# Exploring novel herbicidin analogues by transcriptional regulator overexpression and MS/MS molecular networking

**DOI:** 10.1186/s12934-019-1225-7

**Published:** 2019-10-15

**Authors:** Yuanyuan Shi, Renjie Gu, Yihong Li, Xinwei Wang, Weicong Ren, Xingxing Li, Lifei Wang, Yunying Xie, Bin Hong

**Affiliations:** 1 NHC Key Laboratory of Biotechnology of Antibiotics, Institute of Medicinal Biotechnology, Chinese Academy of Medical Sciences & Peking Union Medical College, No.1 Tiantan Xili, Beijing, 100050 China; 2 CAMS Key Laboratory of Synthetic Biology for Drug Innovation, Institute of Medicinal Biotechnology, Chinese Academy of Medical Sciences & Peking Union Medical College, No.1 Tiantan Xili, Beijing, 100050 China

**Keywords:** Regulator *hcdR2*, Molecular networking, Herbicidin analogues

## Abstract

**Background:**

Herbicidin F has an undecose tricyclic furano-pyrano-pyran structure with post-decorations. It was detected from *Streptomyces mobaraensis* US-43 fermentation broth as a trace component by HPLC–MS analysis. As herbicidins exhibit herbicidal, antibacterial, antifungal and antiparasitic activities, we are attracted to explore more analogues for further development.

**Results:**

The genome of *S. mobaraensis* US-43 was sequenced and a herbicidin biosynthetic gene cluster (*hcd*) was localized. The cluster contains structural genes, one transporter and three potential transcription regulatory genes. Overexpression of the three regulators respectively showed that only *hcdR2* overexpression significantly improved the production of herbicidin F, and obviously increased the transcripts of 7 structural genes as well as the transporter gene. After performing homology searches using BLASTP in the GenBank database, 14 *hcd*-like clusters were found with a cluster-situated *hcdR2* homologue. These HcdR2 orthologues showed overall structural similarity, especially in the C-terminal DNA binding domain. Based on bioinformatics analysis, a 21-bp consensus binding motif of HcdR2 was detected within 30 promoter regions in these genome-mined clusters. EMSA results verified that HcdR2 bound to the predicted consensus sequence. Additionally, we employed molecular networking to explore novel herbicidin analogues in *hcdR2* overexpression strain. As a result, ten herbicidin analogues including six new compounds were identified based on MS/MS fragments. Herbicidin O was further purified and confirmed by ^1^H NMR spectrum.

**Conclusions:**

A herbicidin biosynthetic gene cluster (*hcd*) was identified in *S. mobaraensis* US-43. HcdR2, a member of LuxR family, was identified as the pathway-specific positive regulator, and the production of herbicidin F was dramatically increased by overexpression of *hcdR2*. Combined with molecular networking, ten herbicidin congeners including six novel herbicidin analogues were picked out from the secondary metabolites of *hcdR2* overexpression strain. The orthologues of herbicidin F pathway-specific regulator HcdR2 were present in most of the genome-mined homologous biosynthetic gene clusters, which possessed at least one consensus binding motif with LuxR family characteristic. These results indicated that the combination of overexpression of *hcdR2* orthologous regulator and molecular networking might be an effective way to exploit the “cryptic” herbicidin-related biosynthetic gene clusters for discovery of novel herbicidin analogues.

## Background

**Fig. 1:**
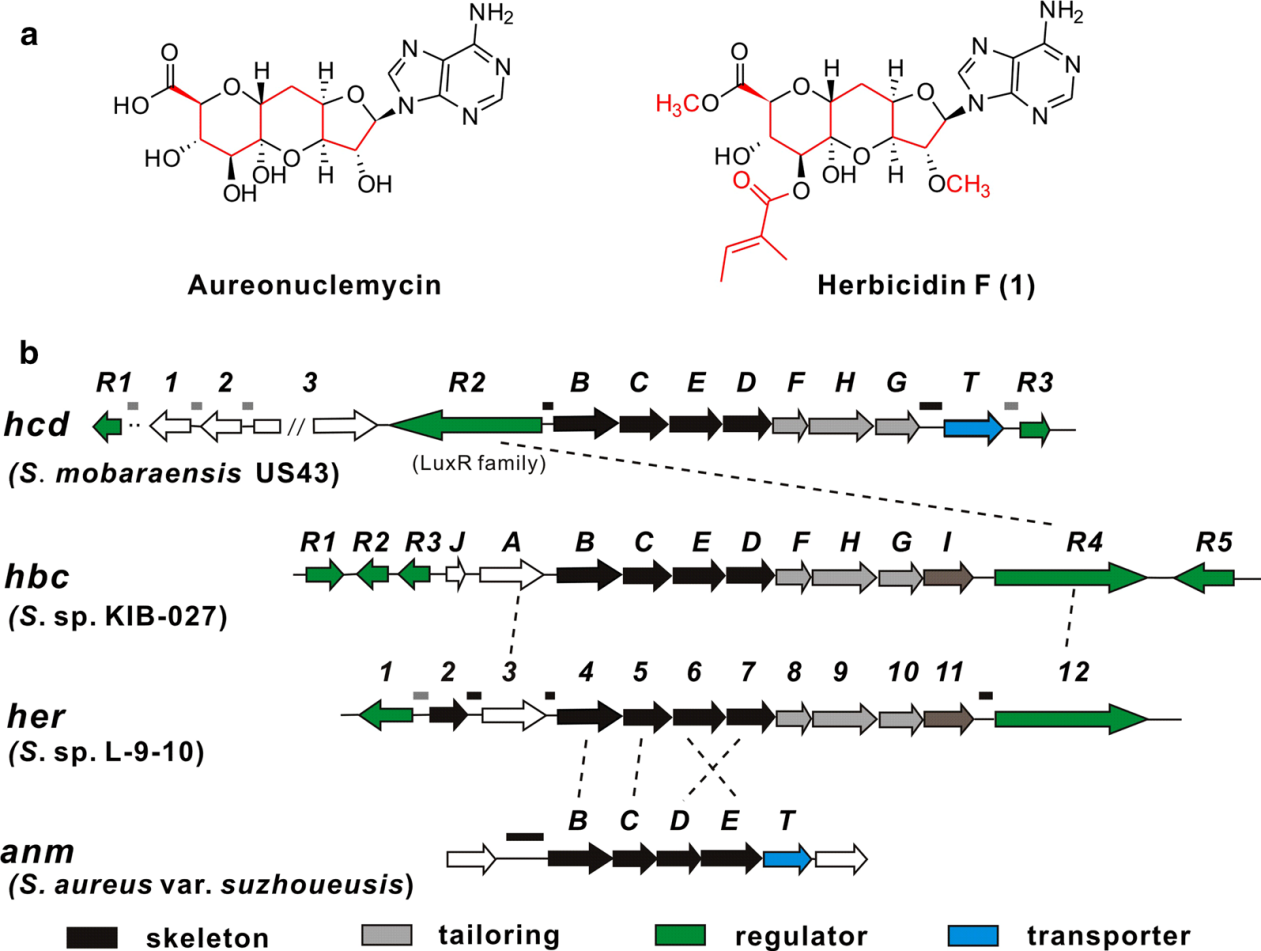
Chemical structures and biosynthetic gene clusters of aureonuclemycin and herbicidin F. **a** The structures of aureonuclemycin and herbicidin F (1). **b** Organization of the *hcd*, *hbc*, *her* and *anm* biosynthetic gene clusters. Lines above the clusters are intergenic regions for FIMO analysis. Black lines contain sequence matching with the consensus binding motif and gray lines don’t have the matched sequence

*Streptomyces mobaraensis* US-43 (former named *S. verticillus* var. *pingyangensis* n. var, CPCC 203575), isolated from a soil sample collected from Pingyang, Zhejiang Province, China, produced a series of glycopeptide antibiotics such as bleomycin analogues [1–3]. Among them, pingyangmycin and boanmycin have been approved by SFDA for cancer treatment in China. As a preserved strain in our laboratory, its secondary metabolites in different fermentation conditions were analyzed to explore new compounds, and piericidin A1 and a group of isocoumarins have been obtained (Additional file 1: Figure S1). Additionally, a trace component was detected by LC–MS and speculated as herbicidin F (**1**) based on UV spectrum and MS/MS fragmentation profile. Herbicidins (Fig. 1) are adenosine-derived nucleoside antibiotics that have a characteristic tricyclic furano-pyrano-pyran structure with different decorations. They have been isolated from *S. saganonensis* [4–7], *S.* sp. L-9-10 [8], *S. scopuliridis* RB72 [9] and *S.* sp. CB01388 [10]. Herbicidins show selective herbicidal activity toward dicotyledonous plants [4], and also exhibit antialgal [7] and antifungal [6] activities. Recently, a report highlights the herbicidin scaffold for anti-*Cryptosporidium* drug development [10]. The complex chemical structures and diverse biological activities have attracted our attention for further exploration of herbicidin congeners and their biosynthesis mechanisms. Although the structure of herbicidin F was reported [6], there were no reports about its biosynthetic gene cluster at the beginning of this work. But at that time a Chinese patent by Tang’s group [11] has demonstrated the minimal gene cluster of aureonuclemycin (Fig. 1), the bare tricyclic core structure of herbicidins, produced by *S. aureus* var. *suzhoueusis*. It contains four necessary genes (*anmB*, *anmC*, *anmD* and *anmE*) by in-frame deletion and heterologous expression, which is reported in a recent paper that elucidated the herbicidin tailoring pathway [12]. Therefore, the four genes responsible for tricyclic core assembly provided important clues to identify herbicidin biosynthetic gene cluster. Here we report the successful mapping and identification of a herbicidin biosynthetic gene cluster (*hcd*) in *S. mobaraensis* US-43 by bioinformatics analysis, which is largely homologous to recently reported *her* in *S.* sp. L-9-10 [13] and *hbc* in *S.* sp. KIB-027 [12] responsible for herbicidin biosynthesis. Furthermore, the pathway-specific regulator was identified by overexpression of the potential regulators located near the *hcd* cluster, and *hcdR2* exerted a significant positive role in the production of herbicidin F, which leads to the acquisition of enough amount of herbicidin F for structural determination by NMR spectra.

After significant improvement of the production of herbicidin F, molecular networking, a computational strategy that assists organization of the tandem MS/MS data [14], was used to identify novel herbicidin congeners in *hcdR2* overexpression strain. Based on the assumption that related structures can produce similar fragment patterns in tandem mass spectra, molecular networking produce an MS/MS spectral similarity map that allows the visualization of structurally related molecules [15], which makes the identification of analogues more efficiently. The workflow of molecular networking, available on the Global Natural Product Social Molecular Networking (GNPS, https://gnps.ucsd.edu/ProteoSAFe/static/gnps-splash.jsp) web site, has been successfully applied in the discovery of novel natural products [16–19]. In our work, after LC–MS/MS analysis of the secondary metabolites of the pathway-specific positive regulator overexpression strain, we employed GNPS for further exploration of herbicidin analogues. Six new herbicidins were firstly reported here by MS/MS analysis. Among them, herbicidin O was purified and further confirmed by proton nuclear magnetic resonance (^1^H NMR) spectrum.

## Results

### Bioinformatics identification of the herbicidin biosynthesis gene cluster (*hcd*) in *S. mobaraensis* US-43

*Streptomyces mobaraensis* US-43, a well-studied strain in our lab, was previously identified to produce a series of bleomycin analogues. Through LC–MS analysis of its fermentation broth, some trace components were detected and a compound (**1**) with UV spectrum and MS/MS fragmentation profile identical to herbicidin F attracted our attention. Among the reported herbicidins, herbicidin F showed better inhibitory activity against *Trychophyton mentagrophytes*, *T. rubrum*, *T. asteroids*, *T. megninii* and some other fungi and markedly non-toxic to animals [4, 6]. As aureonuclemycin was identified to bear the bare nucleoside core of herbicidin F (Fig. 1a), its necessary biosynthesis genes (*anmB*, *anmC*, *anmD* and *anmE*) [11, 12] (Fig. 1b) were used as targets to explore the possible herbicidin gene cluster. The draft genome of *S. mobaraensis* US-43 was sequenced (GenBank Accession No. VOKX00000000, Additional file 1: Figure S1). By BLASTP and antiSMASH analysis of the genome of *S. mobaraensis* US-43 (Additional file 1: Table S1), there was only one predicted cluster containing four correspondingly homologous genes in one operon (Fig. 1b). Downstream of the four core genes, there are two methyltransferase genes which were speculated to be involved in the methylation of herbicidin F. Upstream, there’s one β-ketoacyl synthase that was first thought might be responsible for the biosynthesis of tiglyl moiety in herbicidin F. One transporter, three transcriptional regulators and some other genes were located nearby. Thus we hypothesized that this gene cluster, named *hcd* cluster, is potentially responsible for the herbicidin F biosynthetic pathway. The organization of *hcd* cluster is shown in Fig. 1b and the proposed function of each ORF is given in Table 1. During the preparation of this manuscript, the herbicidin biosynthetic gene clusters in *S.* sp. L-9-10 [13] and *S.* sp. KIB-027 [12] were reported in succession (Fig. 1b). In our cluster, *hcdB *~ *H* is homologous to both *her4 *~ *10* and *hbcB *~ *H*. Two genes, *hbcI/her11* encoding a cytochrome P450 monooxygenase and *hbcA/her3* encoding a ketoacyl-ACP synthase III (KAS III) were absent in the *hcd* cluster. The transporter HcdT is of major facilitator superfamily which is absent in *her* and *hbc* cluster (Fig. 1b). There are three transcriptional regulators situated nearby, and among them, *hcdR1* and *hcdR3* encode regulators belong to SARP (*Streptomyces* antibiotic regulatory protein) family while HcdR2 (with its homologues in *her* and *hbc* clusters) is classified in LuxR family (Table 1).

**Table 1:** Annotation and predicted function of genes in *hcd* cluster

Gene	Name	Size (aa)	Identities	BLAST annotation
*smo5214*	*hcdR3*	261		Transcriptional regulator, SARP family
*smo5216*	*hcdT*	503		Major facilitator transporter
*smo5217*	*hcdG*	367	*her10* (68.83%)	*O*-Methyltransferase
*smo5218*	*hcdH*	540	*Her9* (685.54%)	Beta-lactamase
*smo5219*	*hcdF*	286	*Her8* (63.50%)	Methyltransferase
*smo5220*	*hcdD*	370	*anmD* (65.26%)/*her7* (74.93%)	Oxidoreductase
*smo5221*	*hcdE*	435	*anmE* (55.30%)/*her6* (71.56%)	Radical SAM domain protein
*smo5222*	*hcdC*	372	*anmC* (52.82%)/*her5* (56.23%)	Oxidoreductase/dehydrogenase
*smo5223*	*hcdB*	558	*anmB* (56.24%)/*her4* (64.43%)	*S*-Adenosine-l-homocysteine hydrolase
*smo5225*	*hcdR2*	930	*her12* (30.43%)	LuxR family transcriptional regulator
*smo5226*	*hcd3*	2613		Beta-ketoacyl synthase
*smo5227*	*hcd2*	348		Polyprenyl synthetase
*smo5228*	*hcd1*	359		Terpene synthase/cyclase metal-binding domain protein
*smo5236*	*hcdR1*	227		SARP family transcriptional regulator

### Identification of the pathway-specific regulator of *hcd* cluster

**Fig. 2:**
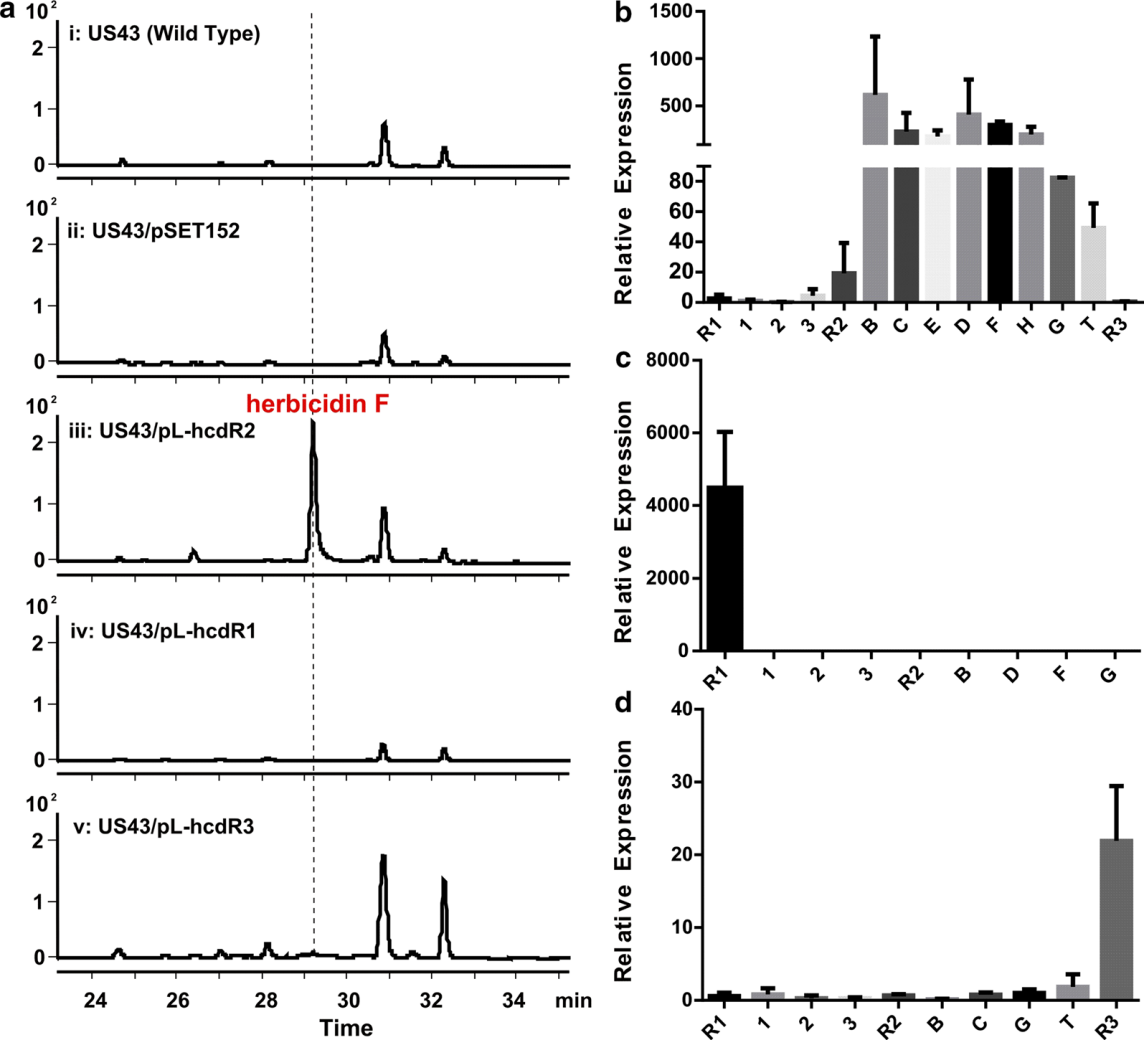
Effects of overexpression of possible regulators on herbicidin production and gene expression. **a** Analysis of the production of herbicidin F in the fermentation broth of different strains by HPLC. i, *S. mobaraensis* US-43; ii, US43/pSET152; iii, US43/pL-hcdR2; iv, US43/pL-hcdR1; v, US43/pL-hcdR3. Transcriptional analysis of different genes in overexpression strains US43/pL-hcdR2 (**b**), US43/pL-hcdR1 (**c**) and US43/pL-hcdR3 (**d**). Data are from three biological samples with two PCR determinations of each. The values were normalized to that of *hrdB* and were represented as mean ± SD. The amounts of each particular transcript in the control strain US43/pSET152 were arbitrarily assigned as 1

As three possible regulators were identified near the predicted *hcd* cluster, we firstly constructed the overexpression strains of each regulator and detected the influence on the production of herbicidin F (**1**) to determine whether the predicted gene cluster was responsible for the biosynthesis of herbicidin F and which one was its pathway-specific regulator. The coding region of each *hcdR* was cloned into the pSET152 [20]-derived expression plasmid pL646 [21] (containing a φC31 *attP*-*int* locus) under the control of a strong constitutive promoter *ermE*p*. The resulting plasmids were respectively introduced into the wild type strain *S. mobaraensis* US-43 by conjugation to give the overexpression strains designated as US43/pL-hcdR1, US43/pL-hcdR2 and US43/pL-hcdR3. The plasmid pSET152 was also transferred to get strain US43/pSET152 as a control. These strains were fermented simultaneously and each of the cultivated broth was analyzed by HPLC. The results showed that only in the strain of US43/pL-hcdR2, the production level of compound **1** was significantly increased by about 20-fold (Fig. 2a), which made the separation and purification of **1** much easier. The strain US43/pL-hcdR2 fermentations were scaled up and 11 mg of compound **1** was obtained. Then it was confirmed as herbicidin F by MS/MS fragments (Additional file 1: Figure S2) and comparison with the reported NMR spectra [8] (Additional file 1: Table S2).

As herbicidin F was structurally determined, the significant increase of herbicidin F production in *hcdR2* overexpression strain suggested that transcription regulator HcdR2 played a positive role in herbicidin F biosynthesis. The gene expression analysis was conducted using quantitative RT-PCR to examine the involvement of the 3 regulatory genes in transcription regulation of genes in *hcd* cluster. The relative level of the transcripts of genes within the cluster were analyzed together with regulators at about 48 h. Compared to control strain US43/pSET152, the transcripts of *hcdB *~ *T* and *hcdR2* were obviously increased in US43/pL-hcdR2, while the transcripts of *hcd1*, *hcd2* and *hcd3* were almost unchanged (Fig. 2b). In US43/pL-hcdR1 and US43/pL-hcdR3, when the overexpressed regulator was upregulated as expected, the transcripts of the above genes have no obvious change compared with that in US43/pSET152 (Fig. 2c, d). These results were consistent with the production level of hebicidin F and confirmed that *hcdR2* is the pathway-specific positive regulator for the biosynthesis of herbicidin F. What is more, the transcript analysis of US43/pL-hcdR2 suggested that the operon *hcdB *~ *H* and *hcdT* is responsible for its biosynthesis. In contrast to our previous prediction of the involvement of Hcd3 (a β-ketoacyl synthase) in the biosynthesis of tiglyl moiety, the transcript analysis results suggested that none of *hcd1 *~ *3* was involved in the biosynthesis of herbicidin F.

### HcdR2 is a conserved pathway-specific regulator for herbicidin production

**Fig. 3:**
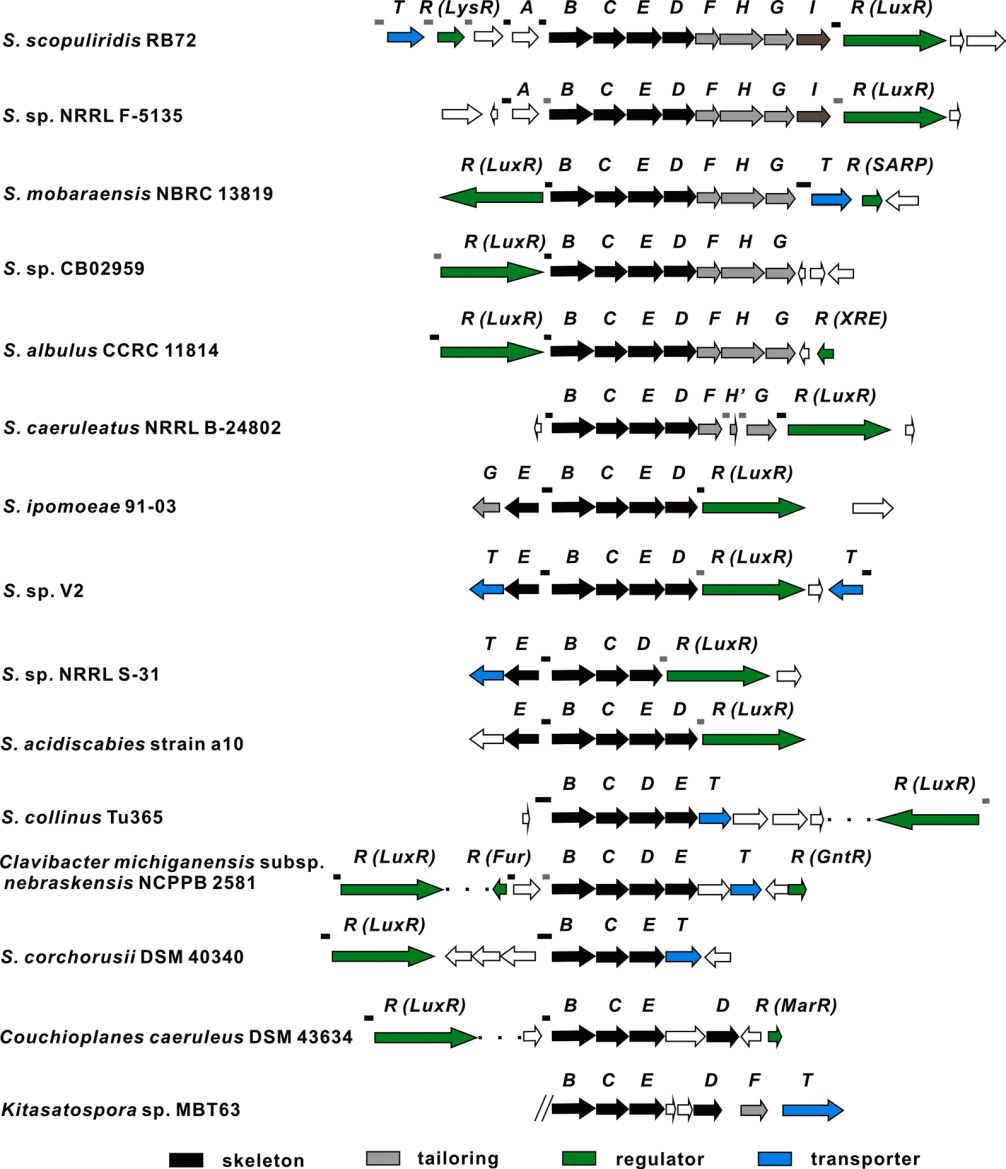
Fifteen more *hcd*-like biosynthetic gene clusters found in NCBI GenBank by genome mining. Lines above the clusters are intergenic regions for FIMO analysis. Black lines contain sequence matching with the consensus binding motif and gray lines don’t have the matched sequence

**Fig. 4:**
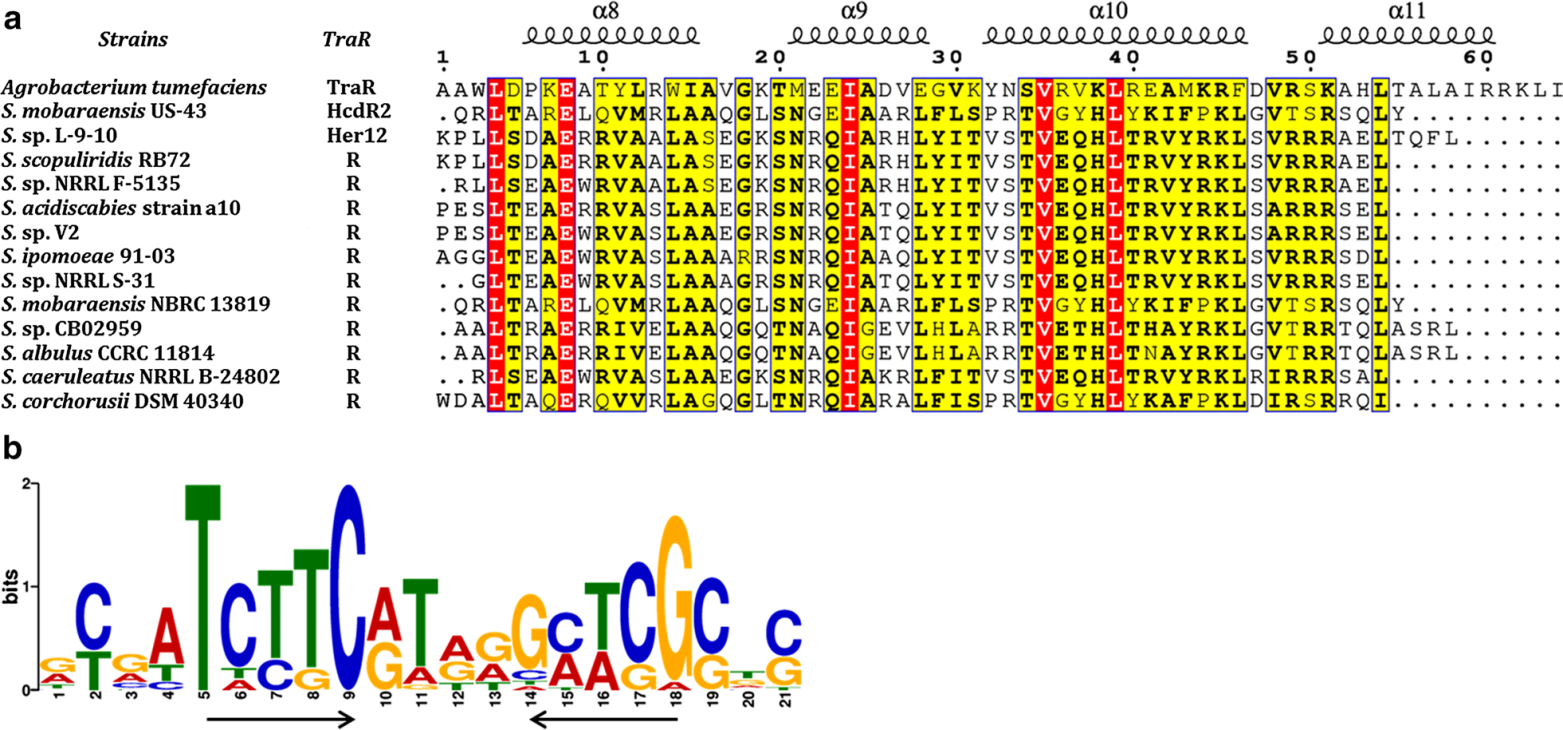
Alignment of DNA binding domain in LuxR regulators and prediction of its consensus binding site. **a** Structure-based alignment of the DNA binding domain (DBD) of HcdR2 with its structurally homologous proteins in different strains. Identities are boxed in red. Similarities are boxed in yellow according to physicochemical properties. Secondary structural elements from the 3D structure of TraR (PDB 1L3L) are displayed on the top of the sequence blocks. GenBank accession numbers of these LuxR regulators are as follows: *S*. sp. L-9-10 (Her12, RYJ25228.1), *S. scopuliridis* RB72 (PVE09951.1), *S.* sp. NRRL F-5135 (WP_078854789.1), *S. acidiscabies* strain a10 (GAQ51936.1), *S.* sp. V2 (PWG13438.1), *S. ipomoeae* 91-03 (EKX64212.1), *S.* sp. NRRL S-31 (WP_079165723.1), *S. mobaraensis* NBRC 13819 (EMF01239.1), *S.* sp. CB02959 (PJN35945.1), *S. albulus* CCRC 11814 (EPY92754.1), *S. caeruleatus* NRRL B-24802 (KUN91385.1), *S. corchorusii* DSM 40340 (KUN18075.1). **b** The predicted HcdR2 consensus binding site identified by MEME Suite. Inverted arrows denote the dyad symmetry

HcdR2 belongs to LuxR family and contains a typical helix-turn-helix (HTH) DNA binding domain (DBD) at the C-terminus. Interestingly, BLASTP analysis in GenBank using *hcdB/C/D/E* as a query showed the existence of 15 more *hcd/hbc/her/anm*-like clusters in actinomycetes (Fig. 3). The majority of them (11 clusters) also have a cluster-situated transcriptional regulator belonging to LuxR family. These regulators, together with Her12, show 30–43% amino acid identity to HcdR2 over the full length of the protein except the regulator in *S. mobaraensis* NBRC 13819 (99.78% amino acid identity). HHpred and BLASTP analyses of these regulators show high 3D structure similarities with conserved domains including an N-terminal AAA ATPase domain and a C-terminal helix-turn-helix (HTH) DNA binding domain (DBD). Furthermore, the alignment of DBD domains of these orthologues of HcdR2 with the closest DBD structural neighbor of LuxR family regulator TraR shows overall homology and displays the domain architecture of a tetrahelical version of the HTH motif (Fig. 4a) using on-line ESPript sever [22] (mean similarity 66.74%). The HTH architecture was responsible for multipolarity and binding specific DNA sites near target promoters to modulate gene expression. Therefore we took each *hcdB*p and other intergenic regions in total 17 clusters (14 mined-clusters plus *hcd*, *her* and *anm*, marked in Figs. 1 and 3, which had 48 sequences in total), except for *hbc* in *S.* sp. KIB-027 (sequence is not available in GenBank) and cluster in *Kitasatospora* sp. MBT63 (sequence of the cluster is not complete), to search the possible HcdR2 consensus binding sequence using the on-line program MEME Suite 5.0.4 [23–25].

Firstly, a possible HcdR2 consensus binding sequence with highest score was identified in 13 promoter regions by MEME-ChIP, a block of MEME Suite. This consensus binding sequence possessed a potential palindromic sequence, consistent with known binding sites of regulators of the LuxR type, such as LuxR [26], TraR [27], LasR [28] and QscR [29]. Then we employed the block FIMO (Find Individual Motif Occurrences) to scan 48 intergenic regions for individual matches to the possible motif matrix. The results showed 30 promoter regions were matched and ranked by *p* value (Figs. 1, 3). Each of the 14 scanned strains together with *hcd*, *her* and *anm* cluster possessed at least one 21-bp consensus binding motif with dyad symmetry (Fig. 4b), the majority of which were located in *hcdB* homologous gene promoter regions. It closely resembled the consensus motifs determined for other LuxR-type regulators, but has a stronger preference for one side of the imperfect palindrome (Fig. 4b, left). These results hinted that orthologues of HcdR2 might regulate the production of herbicidin analogues by binding the consensus DNA sequence in different strains. There are four clusters with the consensus binding sites but without the homologue of HcdR2 nearby, however, in three of them a highly conserved HcdR2 regulator may be found elsewhere in the whole genome. Interestingly, *anm* also has the consensus binding site at the promoter region of *anmB*. Although the genome sequence of aureonuclemycin-producing strain (*S. aureus* var. *suzhoueusis*) is not available, some genome-mined clusters without herbicidin tailoring genes also have the HcdR2 homologues within their clusters. These results suggest the pathway-specific regulatory mechanism is conserved in different strains harboring *hcd/hbc/her/anm*-like clusters.

**Fig. 5:**
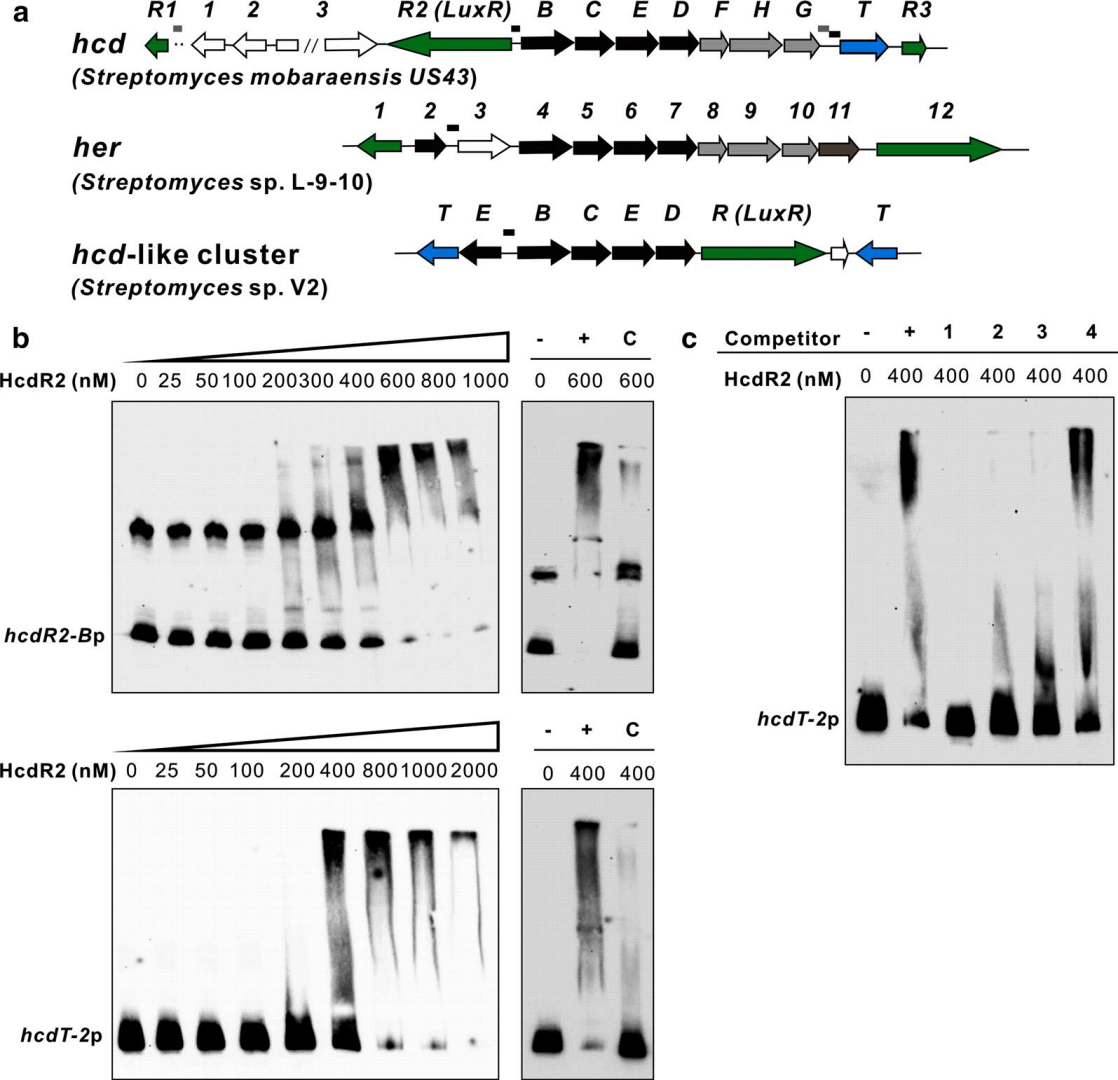
EMSA analysis of HcdR2 with the postulated promoter regions of the *hcd*-like clusters. **a** Three *hcd*-like clusters with promoter regions analyzed in EMSA experiments. Black lines above the ORFs are DNA fragments containing HcdR2 consensus binding motif(s), and gray lines are DNA fragments containing no consensus binding motif. **b** EMSA assays of 5′ biotin-labeled fragments *hcdR2*-*B*p and *hcdT*-*2*p with purified HcdR2. The minus indicates probe only, and the plus indicates probe incubated with HcdR2 at a certain concentration. C indicates probe incubated a certain concentration of HcdR2 with a 200-fold excess of unlabeled specific competitor DNA fragment. **c** Competition reactions of 5′ biotin-labeled *hcdT*-*2*p probe incubated with 400 nM HcdR2. 200-fold excess of unlabeled competitor DNA fragments were added respectively. 1–4 represent *hcdT*-*2*p, *her3*p from *Streptomyces* sp. L-9-10, *B*p from *Streptomyces* sp. V2 and *hcdR1*p respectively

To verify whether HcdR2 binds to the predicted DNA sequence, we performed a series of electrophoretic mobility shift assays (EMSAs). HcdR2 was overexpressed in *E. coli* BL21(DE3) as a His_10_-tagged protein with a predicted molecular mass of 100,983 Da, and purified by nickel affinity chromatography (Additional file 1: Figure S3A). The EMSA results showed that the divergent intergenic region fragment *hcdR2*-*B*p (containing two consensus binding motifs) and *hcdT* upstream region fragment *hcdT*-*2*p (containing a consensus binding motif) were bound by HcdR2 specifically, as shifting of the probes was decreased when the excess unlabeled specific competitor DNA fragments were added into the binding reactions (Fig. 5b). No shifting of the probe occurred when *hcdT*-*1*p fragment containing no consensus binding motif was analyzed (Additional file 1: Figure S3B). When two unlabeled competitor DNA fragments, *her3*p from *Streptomyces* sp. L-9-10 and *B*p from *Streptomyces* sp. V2, were added in excess to binding reactions, shifting of the probes was decreased (Fig. 5c, lanes 2 and 3), which strongly suggested HcdR2 might bind specifically to these two DNA fragments containing a consensus binding motif respectively. Consistent with the motif prediction, an excess of *hcdR1*p containing no consensus binding motif didn’t decrease the shifting of the probe (Fig. 5c, lane 4). These results adequately demonstrate that the predicted promoter regions with consensus binding motif (Fig. 3) are regulated by HcdR2. This warranted a reliable strategy to activate newly genome-mined *hcd/hbc/her/anm*-like clusters by overexpression of HcdR2 or its orthologue in their own cluster.

### New herbicidin analogues were discovered from *hcdR2* overexpression strain by molecular networking

**Fig. 6:**
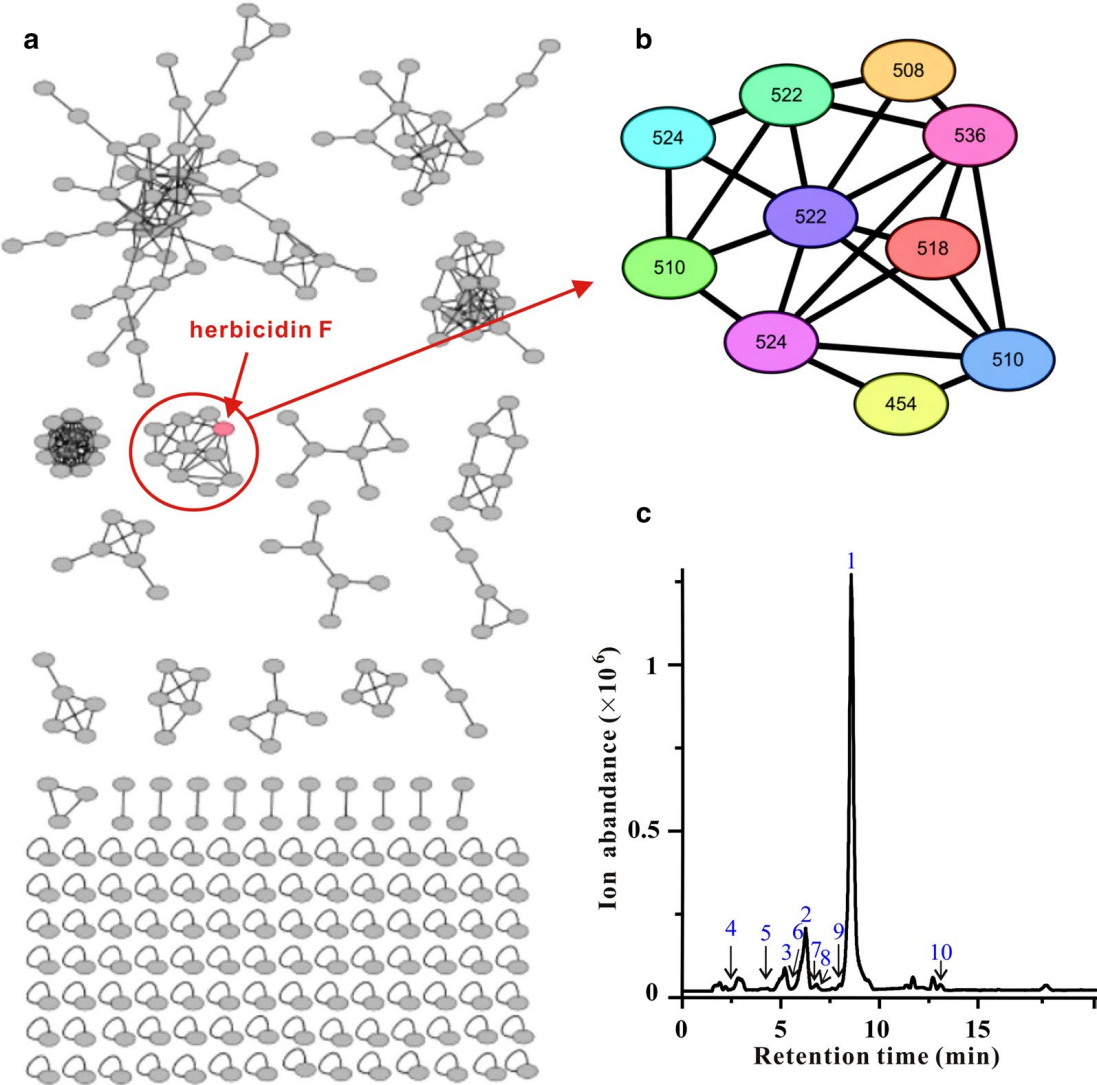
Molecular networking directed discovery of new herbicidin analogues. **a** Molecular network consisting of all parent ions detected by LC–MS in the extract crude of *hcdR2* overexpression strain. **b** A constellation for potential herbicidins was picked out using herbicidin F as a probe and amplified for displaying. This constellation has ten nodes possessing precursor ions ranging from *m/z* 508 to 536 [M+H]^+^ (Node labels show the precursor masses). **c** Based on the molecular networking results, the ten herbicidin peaks (**1**–**10**) corresponding to the ten nodes were found in the LC–MS spectrum

**Fig. 7:**
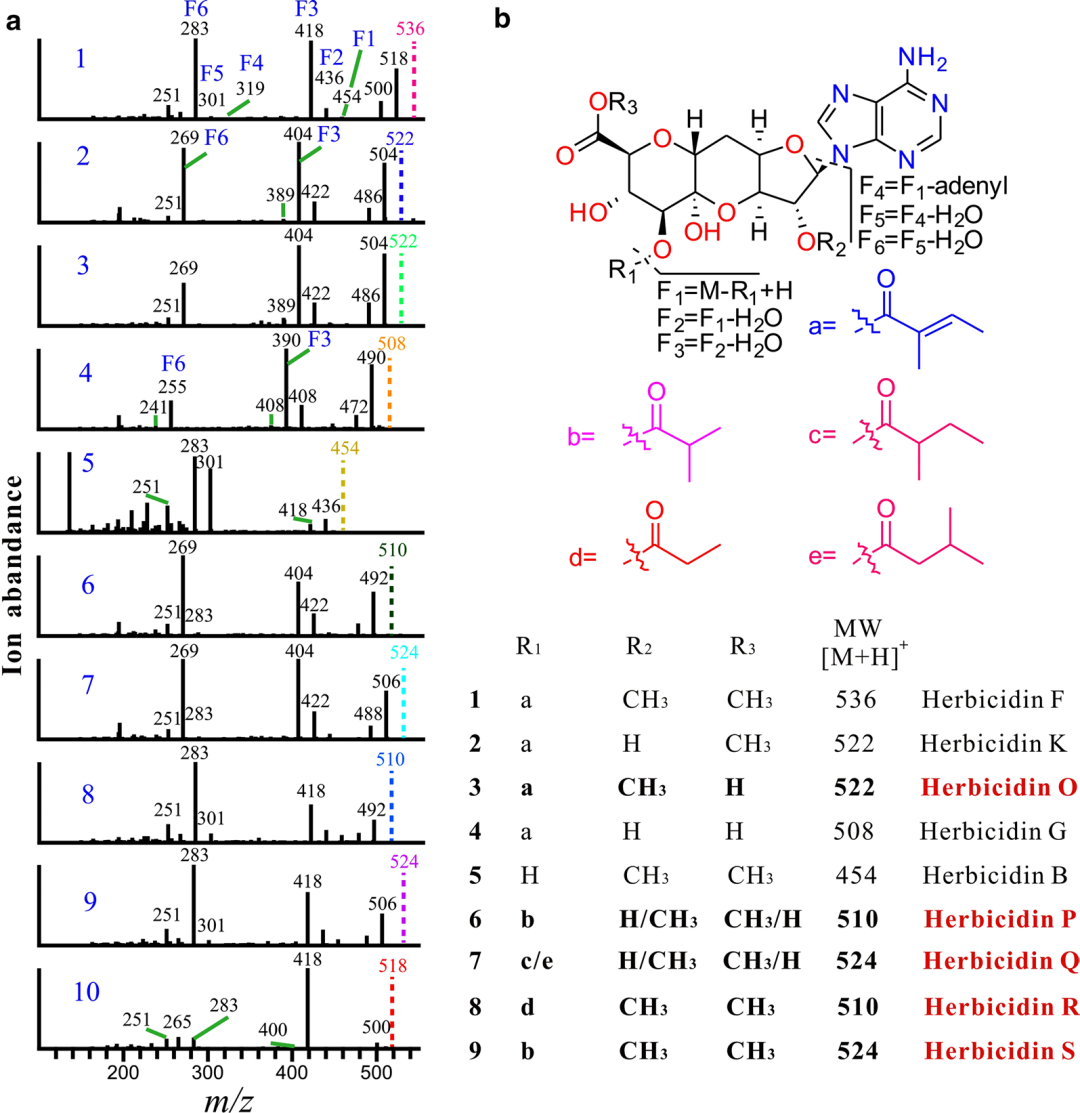
ESI(+)–MS/MS data and structures of ten herbicidin analogues. **a** MS/MS analysis for compounds **1**–**10**. The parent ions are indicated in dotted line and the same color as that of the corresponding nodes in GNPS. The diagnostic fragments are indicated. **b** The tentative structures of compounds **1**–**9**. The potential new herbicidin analogues are highlighted in red color

With the dramatically increased production of herbicidin F in *hcdR2* overexpression strain, some trace herbicidin congeners that were undetectable in the wild type strain were discovered. As molecular networking is a powerful tool to visualize the structurally related molecules [14, 15], we employed it to analyze herbicidin congeners in the fermentation broth of *hcdR2* overexpression strain. The crude extract of US43/pL-hcdR2 fermentation broth was first analyzed on an Agilent 1200 instrument (Agilent Technologies, Santa Clara, CA, USA) coupled to an LTQ XL ion trap mass spectrometer. The LC–MS/MS data were uploaded to MassIVE server (massive.ucsd.edu) and analyzed using a GNPS based molecular networking workflow to generate molecular networks. The resulting spectral networks were visualized using Cytoscape V3.5.1 [30], where nodes represented precursor masses. A subnetwork containing the node corresponding to herbicidin F was identified in the whole molecular network from the crude extract of US43/pL-hcdR2 (Fig. 6). This constellation contained ten nodes possessing precursor ions ranging from *m/z* 508 to 536. Detail analysis of their LC–ESI(+)MS (Fig. 6) and ESI(+)–MS/MS spectra (Fig. 7a) resulted in identification of four known herbicidins (**1**, **2/3**, **4**, and **5**) and six potential new herbicidin structures (**3/2**, **6**–**10**) (Fig. 7b).

Compound **1,** herbicidin F, has a molecular weight of 536 and characteristic MS/MS fragmentation patterns (Additional file 1: Figure S2 and Fig. 7). In the ESI(+)–MS/MS spectrum of compound **1**, fragments *m/z* 418 (F3) and 283 (F6) are the main peaks corresponding to [M-tigly-2H_2_O + H]^+^ and [M-tigly-adenyl-2H_2_O + H]^+^, respectively, and can serve as the diagnostic daughter ions. Fragments *m/z* 518, 500, 454 (F1), 436 (F2), 319 (F4) and 301 (F5), corresponding to [M-H_2_O + H]^+^, [M-2H_2_O + H]^+^, [M-tigly + H]^+^, [M-tigly-H_2_O + H]^+^, [M-tigly-adenyl + H]^+^, and [M-tigly-adenyl-H_2_O + H]^+^, respectively, together with the main fragments F3 and F6 constitute the characteristic MS/MS spectral profile of herbicidins.

**Fig. 8:**
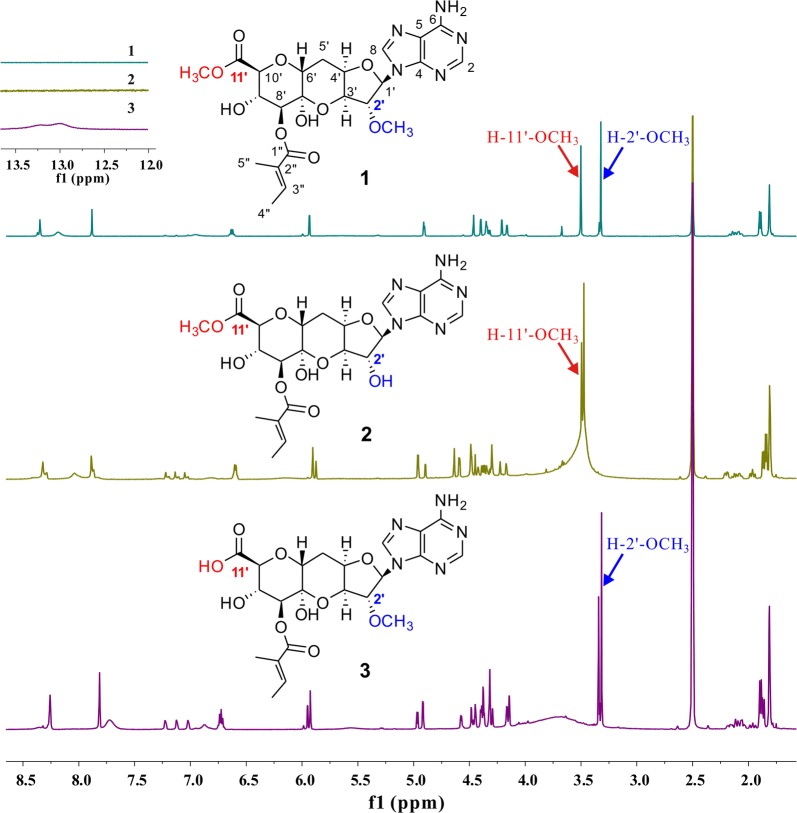
The comparison of ^1^H NMR spectra for 1, 2 and 3 (in DMSO-*d*_*6*_)

Compounds **2** and **3** showed the similar MS/MS patterns with herbicidin F (**1**). Furthermore, compound **2** has the same quasi-molecular ion and fragments as compound **3**, 14 Da less than those of herbicidin F (**1**) (Fig. 7), suggestive of the absence of a methyl at the position R_2_ or R_3_. To further characterize their structures, the compounds **2** (1 mg) and **3** (0.5 mg) were purified. Both of **2** and **3** exhibited the characteristic UV spectrum of nucleoside, the maximum absorbance at approximate 260 nm (Additional file 1: Figure S4). Based on the high resolution electrospray ionization mass spectrometry (HR-ESIMS) [M + H]^+^ *m/z* 522.1857 (calcd for C_22_H_28_O_10_N_5_, *m/z* 522.1836), compound **2** and **3** are determined to have the same molecular formula of C_22_H_27_O_10_N_5_, a CH_2_ less than that of herbicidin F, which further confirmed the above speculation of the absence of methyl at the position R_2_ or R_3_. The position of the methyl was further determined by ^1^H NMR spectrum. The ^1^H NMR spectra of compounds **2** and **3** were collected in DMSO-*d6* to obtain the hydroxyl proton signals which can assist to assign the position of methyl. In DMSO-*d6*, both compounds had two comparable sets of ^1^H NMR signals (appr. 1:0.7 for **2** and 2:3 for **3**, Additional file 1: Figures S5, S6). This phenomenon arose from the equilibrium between hemiketal and free carbonyl forms of herbicidins, which had also been observed for 11′-*O*-demethylherbicidin A and 11′-*O*-demythylherbicidin B in D_2_O according to the literature [13]. To be convenient for comparison, the solvent was switched to DMSO-*d*_*6*_ for compound **1**. Comparing the ^1^H NMR spectrum of **2** with that of **1** (Fig. 8) revealed the absence of H-2′-OCH_3_ signal (*δ*3.32 (s, 3H)/3.34 (s, 3H)) in the former, which confirmed that **2** is short of a methyl at the position R_2_ and has the same structure as herbicidin K. Comparing the ^1^H NMR spectrum of **3** with those of **1** and **2** (Fig. 8) indicated the loss of H-11′-OCH_3_ signal (*δ*3.50 (s, 3H)/3.67 (s, 3H)) and the presence of H-11′-COOH signal (*δ*13.00 (s, 1H) in the former, which confirmed that **3** is short of a methyl at the position R_3_, thus a new herbicidin F analogue bearing a free carboxyl group at C-11′, which was named as herbicidin O.

The total or partial structures of compounds **4**–**10** were tentatively deduced by comparison of their MS/MS fragments with those of **1**–**3** (Fig. 7). Compounds **4** and **5** were determined to be herbicidin G and B, according to the quasi-molecular ions (28 Da and 82 Da less than that of **1**, respectively) and diagnostic fragments. Compounds **6** and **7** exhibited the same F3 ([M-tigly-2H_2_O + H]^+^, *m/z* 404) and F6 ([M-tigly-adenyl-2H_2_O + H]^+^, *m/z* 269) fragments as compounds **2** and **3**, suggesting that compounds **6** and **7** differ from **2** and **3** in R_1_ substituents. The quasi-molecular ion of **7** [M+H]^+^ at *m/z* 524 was 2 Da more than those of **2** and **3**, indictive of the reduction of the double bond in tigly group and the probable substitute of tigly group in **2** with isovaleryl or 2-methylbutyryl group in **7**. The molecular weight of **6** showed 14 Da less than that of **7**, suggestive of the shortage of a methyl group and the presence of isobutyryl group at R_1_ of **6,** the same as the substituent of herbicidin E. Due to the small amounts of compounds obtained, the position of methyl at R_2_ or R_3_ in **6** and **7** are not determined. Compounds **8** and **9** have the same F3 ([M-tigly-2H_2_O + H]^+^, *m/z* 418) and F6 ([M-tigly-adenyl-2H_2_O + H]^+^, *m/z* 283) fragments as herbicidin F (**1**), indictive of the only difference between them in R_1_ substituents. The molecular mass of **8** showed 26 Da less than that of herbicidin F, suggesting that **8** should possess a propionyl group at R_1_. The quasi-molecular ion of **9** [M+H]^+^ at *m/z* 524, 12 Da less than that of herbicidin F, indicating that **9** might possess an isobutyryl group at R_1_, the same as the substituents of herbicidin E and compound **6**. Compound **10** exhibited molecule ion [M+H]^+^ at *m/z* 518, 18 Da less than herbicidin F, and the same diagnostic fragments F3 ([M-tigly-H_2_O + H]^+^, *m/z* 418) and F6 ([M-tigly-adenyl-H_2_O + H]^+^, *m/z* 283) as herbicidin F (**1**), indicative of the loss of H_2_O molecule in compound **10**. Although the structures of these congeners can’t be determined undoubtedly due to the trace amounts of compounds, the combination of molecular networking and manual analysis of MS/MS gave a valuable clue to the diversity of herbicidin variants. Compounds **1**–**10** are mainly diversified in R_1_ substituents, which can be tigly, propionyl, isobutyryl, and 2-methylbutyrl/isovaleryl.

## Discussion

In this work, a herbicidin biosynthetic gene cluster (*hcd*) was identified in *S. mobaraensis* US-43 by bioinformatics analysis. The seven structural genes are homologous to the two reported clusters *her* and *hbc*. In these clusters, multiple regulators were present in each cluster (Fig. 1b), which brings a question which one is the pathway-specific regulator for herbicidin biosynthesis. HcdR2, belonging to LuxR family, was identified as the positive pathway-specific regulator by its overexpression and then improvement of the production level of herbicidin F by about 20-fold, which makes it easier to isolate and identify herbicidin F and its congeners. What’s more, 15 more *hcd/hbc/her/anm*-like clusters were found in NCBI GenBank by genome mining, most of which contained one LuxR-type regulator situated in the cluster. These regulators showed similarities in 3D structure, especially in the C-terminal DNA binding domain and N-terminal AAA ATPase domain. As expected, a consensus binding sequence of HcdR2 was detected in the intergenic regions in all of the clusters by bioinformatics analysis, at least one in each cluster. Although this 21-bp consensus motif exhibits dyad symmetry, HcdR2 showed a unique characteristic with the less conserved sequence on the right side of palindrome, which probably results from the structural differences in the HcdR2-like proteins (Fig. 4b). Furthermore, the EMSA results confirmed that the promoter regions containing the consensus binding motif were regulated by HcdR2 or its orthologues. Therefore, we speculated these HcdR2-like regulators are conserved in *hcd/hbc/her/anm*-like clusters and play a positive role in the biosynthesis of herbicidin/aureonuclemycin congeners by binding consensus DNA sequence, which providing a strategy for activating novel *hcd/hbc/her/anm*-like clusters to discover and identify more herbicidin/aureonuclemycin analogues.

The transcription analysis of predicted genes showed that HcdB-H and HcdT are responsible for the biosynthesis of herbicidin F. Compared with *her* and *hbc*, transporter is unique in *hcd* and responsible for herbicidin transportation. The seven structural genes are homologous to both *her4 *~ *10* [13] and *hbcB *~ *H* [12]. According to the recently reported characterization of the biosynthetic pathway for herbicidins, we speculated that the biosynthesis of herbicidin F was firstly catalyzed by HcdB/C/D/E for core assembly, then the serine hydrolase (HcdH) for tiglyl loading and last two steps of SAM-dependent methylation (HcdF/G). Because of the lack of *hbcI/her11* encoding a cytochrome P450 monooxygenase in *hcd*, which catalyzed the hydroxylation reaction on tiglyl moiety, no compounds have been found with hydroxylation on acyl group (R_1_) in *S. mobaraensis* US-43 so far. Unlike our original prediction, none of Hcd1/2/3 was related to the biosynthesis of the tiglyl moiety to the core of the herbicidins according to the transcriptional analysis. Recently, Lin et al. [13] speculated that the biosynthesis of the tiglyl moiety follows a pathway similar to what is observed in plant, which might be also present in *S. mobaraensis* US-43. A recent report by Tang’s group reported that HbcH catalyzed the transfer of tiglyl-CoA to form herbicidins by in vivo disruption and in vitro enzymatic assays [12]. Furthermore, the substrate spectrum of acyltransferase HbcH was also investigated in vitro and many acyl groups can be transferred to form a series of derivatives in their study [12]. Here, the six newly identified herbicidin congeners in fermentation broth of HcdR2 overexpressed *S. mobaraensis* US-43 were diverse in acyl groups including propionyl, isobutyryl and 2-methylbutyryl/isovaleryl, which was consistent with their in vitro result and indicated that the substrate flexibility of the serine hydrolase (HcdH) was a useful feature for generating new herbicidin analogues.

A 21-bp consensus binding sequence of HcdR2 was detected using the on-line program MEME Suite. The results showed 30 promoter regions matched and each of the 17 scanned strains possessed at least one consensus binding site (Figs. 1, 3). These clusters can be divided into 2 groups. One only contains genes for the bare tricyclic core assembly similar to *anm* and the other group has additional tailoring genes similar to *hcd*. In our cluster, there are two predicted binding sites situated between *hcdR2* and *hcdB* and one in *hcdT* promoter region respectively (Fig. 1), which were confirmed to be bound by HcdR2 through a series of EMSAs. No binding site was discovered in *hcdR1, hcdR3, hcd1* and *hcd2* promoter regions, which was consistent with the transcription analysis of HcdR1 ~ 3 in overexpression strains. All of the promoter regions of *hcdB* and its homologue occupied a binding site except in the clusters from *S*. sp. NRRL F-5135 and *Clavibacter michiganensis* subsp. *nebraskensis* NCPPB 2581. In these two clusters, there is a consensus binding site existing in the promoter region of the upstream gene in the same direction as in clusters of *S.* sp. L-9-10 and *S. scopuliridis* RB72. Except for the upstream gene in *Clavibacter michiganensis* subsp. *nebraskensis* NCPPB 2581, the other three upstream genes are homologous to *hbcA*. HbcA was originally thought to catalyze the esterification of –OH at C-8′, but it was confirmed not involved in this reaction later [12]. Here the promoter region of *her3* (homologous to *hbcA*) was found to be bound by HcdR2 (Fig. 5c, lane 2), which hint HbcA/Her3 may be somehow related to the biosynthesis of herbicidin analogues which have not be identified. In addition, there were some binding sites located in the promoter regions of HcdR2-like regulators, suggesting this regulator may control the expression of itself, possibly involved in the feedback regulation of the herbicidin production. Several transporter genes also had this consensus binding site in their promoter region. Among them, transporters in *anm*-like clusters have similarity with AnmT and belong to MFS superfamily, which may be conserved in *anm* cluster. Besides, a few of new genes were present in the genome-mined clusters and the prediction of the binding sites showed they could express along with other genes, indicating that many novel analogues with more diversity are yet to be discovered. This will be useful for identification and characterization of new biosynthetic parts or modules for herbicidins/aureonuclemycin analogues and lay a foundation for the applications of synthetic biology.

Here, based on the dramatical improvement of the expression of herbicidin gene cluster, we employed molecular networking to analyze the secondary metabolites of *hcdR2* overexpression strain. As a result, herbicidin F and nine other compounds formed a subcluster in the network, and then six new herbicidin congeners were identified by MS/MS spectral analysis. Among them, several congeners were trace components and hard to be distinguished by manual, while they can be easily picked out by automatic molecular networking. In addition, the MS/MS data of herbicidin F in this research has been uploaded to the GNPS library, which will assist GNPS users to find herbicidin congeners from crude extract even if there are no references of herbicidins at hand. Nowadays, with the number of microbial genome sequences growing rapidly, much more *hcd/hbc/her/anm*-like clusters might be discovered by genome mining. Combining with molecular networking, the overexpression of HcdR2 or its orthologue will facilitate the exploitation of novel herbicidins.

## Conclusions

In this study, a herbicidin biosynthetic gene cluster (*hcd*) was identified in *S. mobaraensis* US-43, a strain known for production of bleomycin analogues. Among three potential regulators, HcdR2, belonging to LuxR family, was identified as the conserved, positive pathway-specific regulator for herbicidin biosynthesis by overexpression and then the analysis of production level of herbicidin F as well as transcription analysis of the cluster. The homologues of HcdR2 are present in most of the genome-mined *hcd/hbc/her/anm*-like clusters. What’s more, at least one 21-bp consensus binding motif of HcdR2 was identified in each cluster, suggesting HcdR2 is conserved for herbicidin/aureonuclemycin production. Combined with molecular networking, ten herbicidin congeners were picked out from the secondary metabolites of *hcdR2* overexpression strain, six new herbicidin analogues were identified by MS/MS spectral analysis, and the structure of herbicidin O was further confirmed by ^1^H NMR spectrum. These results indicated that the combination of *hcdR2* overexpression and molecular networking is an effective way to activate cryptic *hcd*-like clusters discovered by genome mining, and lay a foundation for the identification of novel herbicidins.

## Materials and methods

### Strains, plasmids and growth conditions

The wild-type *S*. *mobaraensis* US-43 and its derivatives used in this study are listed in Table 2. The wild-type *S*. *mobaraensis* US-43, isolated from the soil of Pingyang, Zhejiang, China, was used as a host strain for the propagation and transformation. *S*. *mobaraensis* US-43 and its derivatives were grown at 28 °C on solid S5 medium [31] for sporulation, on mannitol soya flour (MS) agar medium [32] for conjugation and in the liquid phage medium [33] for isolation of genomic DNA. Herbicidin F was produced with two stage liquid state fermentation. The liquid seed fermentation medium (0.3% high nitrogen corn starch powder, 2% soybean powder, 2.5% glucose, 2% starch, 2% maltose, 0.2% K_2_HPO_4_, and 0.3% NaCl) and fermentation medium (the same as seed medium) were used in the first and secondary fermentation. *Escherichia coli* DH5α [34] was used as a host for general cloning experiments. *E*. *coli* ET12567/pUZ8002 [35] was used for conjugal transfer according to the established protocol [32]. *E*. *coli* strains were incubated in Luria–Bertani medium (LB) [34] at 37 °C. When required, strains were incubated with apramycin (Am, 50 μg/mL), ampicillin (Amp, 100 μg/mL), kanamycin (Km, 50 μg/mL) and chloramphenicol (Cm, 25 μg/mL).

**Table 2:** Strains and plasmids used in this study

Strains/plasmids	Relevant characteristics	References
Strains		
*Streptomyces mobaraensis* US-43	Wild-type strain (herbicidin F-producing strain)	Our laboratory
US-43/pL-hcdR1	*S*. *mobaraensis* US-43 with the expression vector pL-hcdR1, Am^r^	This work
US-43/pL-hcdR2	*S*. *mobaraensis* US-43 with the expression vector pL-hcdR2, Am^r^	This work
US-43/pL-hcdR3	*S*. *mobaraensis* US-43 with the expression vector pL-hcdR3, Am^r^	This work
* Escherichia coli*		
DH5α	General cloning host	[34]
ET12567/pUZ8002	Donor strain for intergeneric conjugation between *E. coli* and *Streptomyces*, Cm^r^, Km^r^	[35]
Plasmids		
pSET152	*Streptomyces* integrative vector, Am^r^	[20]
pL646	pSET152 derivative containing the constitutive promoter *ermE*p*, Am^r^	[21]
pL-hcdR1	pL646 derivative plasmid containing 684 bp complete coding region of *hcdR1*, Am^r^	This work
pL-hcdR2	pL646 derivative plasmid containing 2793 bp complete coding region of *hcdR2*, Am^r^	This work
pL-hcdR3	pL646 derivative plasmid containing 786 bp complete coding region of *hcdR3*, Am^r^	This work
pET16b-HcdR2	pET16b derivative plasmid containing 2793 bp complete coding region of *hcdR2*, Amp^r^	This work

Am^r^, apramycin resistance; Km^r^, kanamycin resistance; Cm^r^, chloramphenicol resistance; Amp^r^, ampicillin resistence

### Construction of *hcdR1*, *hcdR2* and *hcdR3* gene overexpression strains

For overexpression of *hcdR1* in *S*. *mobaraensis* US-43, the complete *hcdR1* gene was amplified using the primer pair pL-hcdR1-F/pL-hcdR1-R in Additional file 1: Table S3. And the PCR product of the *hcdR1* gene was cloned into the *Nde*I-*Bam*HI sites of pL646 [21], a pSET152 [20] -derived expression plasmid with a strong constitutive promoter *ermE*p* in the upstream of the multiple cloning sites. With the same strategy, the *hcdR2* and *hcdR3* gene were cloned into the *Nde*I–*Bam*HI and *Nde*I–*Xba*I sites, respectively. The resulted recombinant plasmid pL-hcdR1, pL-hcdR2 and pL-hcdR3 were introduced into *E. coli* ET12567/pUZ8002 and then transferred into *S. mobaraensis* US-43 by conjugation respectively. The plasmid pSET152 [20] was transferred to *S. mobaraensis* US-43 as controls.

### Analysis of herbicidin F production

*Streptomyces mobaraensis* US-43 wild type and the mutants were cultured on solid S5 medium at 28 °C for 7 days. The spores of *S. mobaraensis* US-43 and the mutants were inoculated in 100 mL seed culture and incubated at 28 °C for 48 h at 220 rpm. Then 5 mL of the resulting culture was seeded into 100 mL of the fermentation medium. This production culture was incubated at 28 °C at 220 rpm for 7 days. The obtained supernatants were analyzed for the production of herbicidin F by LC–MS. For analyzing the analogues, the supernatant of fermentation broth was enriched by Sep-Pak C_18_ Classic Cartridge (Waters Associates), eluted with 50% and 100% methanol solution. HPLC was performed using a C18 column (Agilent, 150 mm × 4.6 mm, 5 μm) with UV detection at 210 nm and 254 nm on an Agilent 1100 instrument (Agilent Technologies, Santa Clara, CA, USA). The samples were eluted with mobile phase CH_3_OH–H_2_O using a flow rate of 1 mL/min: 0–5 min, 5% CH_3_OH; 5–45 min, 5–100% CH_3_OH; 45–55 min, 100% CH_3_OH; 56–60 min, 5% CH_3_OH.

### Transcriptional analysis by real-time RT-PCR (qRT-PCR)

Mycelia of *S. mobaraensis* US-43 grown in fermentation medium for 48 h were collected and frozen in liquid nitrogen. RNA was extracted using the TRIzol reagent according to the protocol (Promega), and treated with DNaseI to remove any contaminating chromosomal DNA. The quantity and purity of the harvested RNA was determined using a NanoDrop 8000 spectrophotometer (Thermo Scientific). 2 μg of each of the total RNA was used as a template for reverse transcription (RT), which was carried out using the *TransScript*^*®*^ One-Step gDNA Removal and cDNA Synthesis SuperMix (Transgen). Gene fragments were amplified from the target genes and detected using the Real-Time PCR Detection System (Bio-Rad). The gene primers used in qRT-PCR reactions are listed in Additional file 1: Table S3. Each reaction mixture was comprised of 12.5 μL of FastStart Universal SYBR^®^ Green Master (ROX) (Roche), 2.5 μL of template, 2.5 μL of forward primer, 2.5 μL of reverse primer and 5 μL of RNase-free H_2_O.

### Bioinformatics analysis

The draft genome of *S. mobaraensis* US-43 was sequenced on a second-generation sequencing platform, Illumina Hiseq 2000, resulting in 1204 Mb data (9,902,314 reads with 463 bp average insert size and about 150-fold average coverage). The genome was assembled into 49 scaffolds, 169 contigs (7,899,533 bp with a GC content of 73.11%) by SOAPdenovo v2.04 [36]. Secondary metabolite biosynthesis gene clusters were predicted by antiSMASH 5.0.0 (Additional file 1: Table S1) [37, 38]. BLASTP was used for genome mining of potential herbicidin/aureonuclemycin clusters using *hcdB/C/D/E* as targets. Every gene in each cluster was blasted and annotated. HHpred and BLASTP were used to analyze the 3D structure and conserved domains. The intergenic regions in each cluster were picked out and submitted to the MEME Suite sever (http://meme-suite.org, motif-based sequence analysis tools) for MEME-ChIP analysis. The locations of the discovered sequence with the highest score in each cluster were collected and submitted for MEME analysis to gain a motif. For further verification of the discovered motif, FIMO analysis was carried out to scan a set of intergenic regions for individual matches to this motif. The *p*-value of a motif occurrence is defined as the probability of a random sequence of the same length as the motif matching that position of the sequence with as a good or better score and it was set to less than 0.001.

### Expression and purification of His_10_-tagged HcdR2

The coding region of *hcdR2* was amplified from *S. mobaraensis* US-43 genomic DNA with primers HcdR2-16b-F2 and HcdR2-16b-R2 (Additional file 1: Table S3), then cloned into pET-16b vector (Novagen) between *Nde*I and *Bam*HI sites, generating the recombinant plasmid pET16b-HcdR2. Then it was transformed into *E. coli* BL21(DE3) for protein expression after authenticated by sequencing. *E. coli* BL21(DE3)/pET16b-HcdR2 were grown in 400 mL LB medium with 100 μg/mL ampicillin at 37 °C to exponential growth phase (OD_600_ of 0.7). IPTG was then added (final concentration 1 mM), and the cultures were incubated at 15 °C for 24 h. The cells were harvested by centrifugation (4000×*g*, 10 min, 4 °C), washed twice with binding buffer (20 mM Na_3_PO_4_, 500 mM NaCl, 20 mM imidazole, pH 7.4), resuspended in 30 mL of the same buffer and lysed by sonication on ice. Cellular debris was removed by centrifugation (12,000×*g*, 20 min, 4 °C). His_10_-tagged HcdR2 was then purified using HisTrap™ HP (GE Healthcare) according to the manufacturer’s instructions, and eluted with elution buffer (20 mM Na_3_PO_4_, 500 mM NaCl, 500 mM imidazole, pH 7.4) using linear gradient. Fractions eluted from the column with 160 mM imidazole were dialyzed against TGEK buffer (50 mM Tris base, 10% glycerol, 1 mM EDTA, 100 mM KCl, pH 8.0) at 4 °C by PD-10 desalting column (GE Healthcare) according to the manufacturer’s instructions, and then stored at − 80 °C. Protein purity was determined by Coomassie Brilliant Blue staining after SDS-PAGE on 8% polyacrylamide gel. The concentration of the purified HcdR2 was determined using Pierce BCA Protein Assay Kit (Thermo Scientific).

### Electrophoretic mobility shift assays (EMSAs)

Promoter fragments were generated by PCR using the primers labeled at their 5′-ends with Biotin (Additional file 1: Table S3) and used as probes in EMSAs. Each 20 μL binding reaction consisted of 2 μL 10× binding buffer (100 mM Tris–HCl, 500 mM KCl, 10 mM DTT, pH 7.5), 20 fmol labeled probe and 25–2000 nM of purified protein. In competition reactions, different unlabeled probes (> 200 fold of the labeled probes) were added respectively. Reactions were incubated at room temperature for 20 min and then run on a native 5% (80:1) acrylamide: bis-acrylamide gel, buffered in 0.5× TBE at 120 V, 4 °C. The gel was then transferred to nylon membrane (Amersham Biosciences) by electrophoretic transfer. The biotin end-labeled DNA was detected by LightShift Chemiluminescent EMSA Kit (Thermo Scientific) according to the manufacturer’s instructions.

### Global natural product social molecular networking (GNPS)

To acquire the LC–MS/MS data for GNPS analysis, the fermentation broth of US43/pL-hcdR2 was enriched using macroporous absorbent resin 4006 column and eluted by 30% and 80% acetone aqueous, respectively. The eluent of 80% acetone was concentrated under pressure, and then was fractioned by flash ODS column. The fractions containing herbicidins were combined to yield the crude extract. Then the crude extract was analyzed on an Agilent 1200 instrument (Agilent Technologies, Santa Clara, CA, USA) coupled to an LTQ XL ion trap mass spectrometer (Thermo Fisher Scientific, Waltham, MA, USA), using a VP-ODS column (150 mm × 4.6 mm, 5 μm, SHIMADZU), with a 1 mL/min, 60 min gradient elution (the same as above). LC–ESI(+)MS/MS data, acquired at a collision energy of 35 eV as .raw file format, were converted to .mzXML file format using MS convert program of ProteoWizard 3.0 and uploaded to MassIVE server (massive.ucsd.edu). The data are analyzed using GPNS molecular networking tool following the instruction provided in the website of https://gnps.ucsd.edu/ProteoSAFe/static/gnps-splash2.jsp. The resulting spectral networks are visualized using Cytoscape version 3.5.1 [30], where nodes represented precursor mass.

### Purification and characterization of compound **1**–**3**

Strain US43/pL-hcdR2 fermentations were scaled up for separation and purification of new analogues. The mycelia were removed by centrifugation, and the supernatant (ca. 4 L) was loaded on a column of macroporous absorbent resin 4006 (400 mL), and after washing with water, the active absorbed materials were eluted with a step gradient elution (30%, 80% and 100% acetone in water) to give three fractions, Fr1 to Fr3. Based on the HPLC analysis results, herbicidin F and their derivatives were found in Fr 2 (1.197 g). Fr2 was separated by reversed-phase flash column chromatography (RediSep column: 40 g C_18_) eluting with 14.8% acetonitrile aqueous containing 0.01% TFA as modifier to afford five subfractions (Fr2-1 to Fr2-5). Fr2-3 (262 mg) was purified by semipreparative HPLC (ReproSil-Pur Basic-C18 column, 5 μm, 250 × 10 mm) eluting with 18% acetonitrile aqueous containing 0.01% TFA as modifier to yield compound **1** (11 mg), **2** (1 mg), **3** (0.5 mg). The samples were analyzed on SHIMADZU LC-20A instrument using ReproSil-Pur Basic-C18 column (5 μm, 150 × 4.6 mm), the same eluent as semipreparative HPLC, and the detector at 254 nm. The HRESIMS data were acquired on Waters^®^ UPLC equipped with Xevo^®^ G2-S QTof. The NMR data were acquired with Bruker spectrometers using DMSO-*d*_*6*_ or CD_3_OD as solvent.

#### ^1^H and ^13^C NMR spectroscopic characterization of **1**

^1^H NMR (600 MHz, CD_3_OD) *δ* 8.36 (s, 1H, H-2), 8.09 (s, 1H, H-8), 6.71 (q, J = 7.1 Hz, 1H, H-3″), 6.09 (d, J = 1.6 Hz, 1H, H-1′), 5.00 (d, J = 3.2 Hz, 1H, H-8′), 4.52 (dd, J = 10.3,5.7, 1H, H-6′) and 4.50 (d, J = 1.8 Hz, 1H, H-3′), 4.45 (s, 1H, H-10′), 4.41 (q, J = 2.4 Hz, 1H, H-4′), 4.30 (dd, J = 3.2, 1.0 Hz, 1H, H-9′), 4.08 (d, J = 1.1 Hz, 1H, H-2′), 3.61 (s, 3H, H-11′-OCH_3_), 3.41 (s, 3H, H-2′-OCH_3_), 2.30–2.22 (m, 2H, H-5′), 1.90 (d, J = 7.0 Hz, 3H, H-4″), 1.85 (s, 3H, H-5″). ^13^C NMR (150 MHz, CD_3_OD) *δ*171.4 (C-11′), 167.2 (C-1″), 154.4 (C-6), 150.3 (C-2), 149.5 (C-4), 142.1 (C-3″), 141.8 (C-8), 128.6 (C-2″), 119.9 (C-5), 93.5 (C-7′), 91.8 (C-2′), 89.1 (C-1′), 79.4 (C-4′), 78.4 (C-10′), 74.7 (C-3′), 72.0 (C-8′), 70.6 (C-9′), 66.7 (C-6′), 58.5 (C-2′-OCH_3_), 52.8 (C-11′-OCH_3_), 26.7 (C-5′), 15.2 (C-4″), 12.5 (C-5″).

#### ^1^H NMR spectroscopic characterization of **1** (mixture of hemiacetal and free carbonyl forms)

^1^H NMR (500 MHz, DMSO) *δ* 8.37 (s, 1H, H-2)/, 8.35 (s, 1H, H-2), 8.19 (s, 2H, H-NH_2_) 7.88 (s, 1H, H-8), 6.63 (qd, *J* = 6.9, 1.2 Hz, 1H, H-3″), 5.93 (d, *J* = 1.9 Hz, 1H, H-1′)/6.00 (d, *J* = 1.6 Hz, 1H, H-1′), 4.91 (d, *J* = 3.1 Hz, 1H, H-8′), 4.46 (s, 1H, H-10′), 4.40 (d, *J* = 2.2 Hz, 1H, H-3′), 4.33 (dd, *J* = 11.6, 5.4 Hz, 1H, H-6′), 4.21 (d, *J* = 1.8 Hz, 1H, H-4′), 4.17 (s, 1H, H-2′) 4.16 (dd, *J* = 3.1, 0.9 Hz, 1H, H-9′), 3.50 (s, 3H, H-11′-OCH_3_)/3.67 (s, 3H, H-11′-OCH_3_), 3.32 (s, 3H, H-2′-OCH_3_)/3.34 (s, 3H, H-2′-OCH_3_), 2.16–2.05 (m, 2H, H-5′), 1.89 (dd, *J* = 7.1, 1.0 Hz, 3H, H-4″), 1.81 (s, 3H, H-5″).

#### ^1^H NMR spectroscopic characterization of **2** (mixture of hemiacetal and free carbonyl forms)

^1^H NMR (600 MHz, DMSO-*d*_*6*_) δ 8.33 (s, 1H, H-2)/8.30 (s, 1H, H-2), 8.04 (s, 2H, H-NH_2_)7.90 (s, 1H, H-8)/7.88 (s, 1H, H-8), 6.61 (dd, *J* = 13.7, 6.8 Hz, 1H, H-3″), 5.91 (s, 1H, H-1′)/5.88 (s, 1H, H-1′), 4.97 (d, *J* = 4.1 Hz, 1H, H-8′)/4.90 (d, *J* = 3.1 Hz, 1H, H-8′), 4.64–4.18 (m, 6H, H-4′, H-6′, H-10′, H-2′, H-3′, H-9′, signals from hemiacetal form and free carbonyl form overlapped with each other), 3.50 (s, 3H, H-11′-OCH_3_)/3.48 (s, 3H, H-11′-OCH_3_), 2.24–1.93 (m, 2H, H-5′), 1.88 (d, *J* = 7.1 Hz, 3H, H-4″)/1.85 (d, *J* = 7.1 Hz, 3H, H-4″), 1.82 (s, 3H, H-5″)/1.81 (s, 3H, H-5″H).

#### ^1^H NMR spectroscopic characterization of **3** (mixture of hemiacetal and free carbonyl forms)

^1^H NMR (500 MHz, DMSO-*d*_*6*_) *δ* 13.00 (s, 1H, H-COOH), 8.26 (s, 1H, H-2), 7.81 (s, 1H, H-8), 7.72 (s, 2H, H-NH_2_), 6.72 (m, 1H, H-3″), 5.95 (d, *J* = 1.9 Hz, 1H, H-1′)/5.92 (d, *J* = 1.9 Hz, 1H, H-1′), 4.97 (d, *J* = 4.1 Hz, 1H, H-8′)/4.92 (d, *J* = 3.1 Hz, 1H, H-8′), 4.57–4.14 (m, 6H, H-4′, H-6′, H-10′, H-2′, H-3′, H-9′, signals from hemiacetal form and free carbonyl form overlapped with each other), 3.34 (s, 3H, H-2′-OCH_3_)/3.32 (s, 1H, H-2′-OCH_3_), 2.20–1.93 (m, 2H, H-5′), 1.89 (dd, *J* = 7.0, 0.9 Hz, 2H, H-4″)/1.87 (dd, *J* = 7.1, 0.8 Hz, 2H, H-4″), 1.81 (d, *J* = 1.0 Hz, 3H, H-5″).

## Supplementary information

**Additional file 1.** Additional tables and figures.

## Data Availability

The data supporting our findings can be found in the main paper and the additional file.
