# Supplementary material for: Exploring novel herbicidin analogues by transcriptional regulator overexpression and MS/MS molecular networking
Source: Microb Cell Fact. 2019 Oct 15;18:175. doi: 10.1186/s12934-019-1225-7 (PMC6794829; doi:10.1186/s12934-019-1225-7)
Supplement: Supplementary file 1 — Additional file 1. Additional tables and figures. [file 12934_2019_1225_MOESM1_ESM.docx]

**Additional material**

**Exploring novel herbicidin analogues by transcriptional regulator overexpression and MS/MS molecular networking**

Yuanyuan Shi^1,2^, Renjie Gu^1^, Yihong Li^2^, Xinwei Wang^2^, Weicong Ren^1^, Xingxing Li^1,2^, Lifei Wang^1,2^, Yunying Xie*^2^, Bin Hong*^1,2^

*^1^ NHC Key Laboratory of Biotechnology of Antibiotics, ^2^ CAMS Key Laboratory of Synthetic Biology for Drug Innovation, Institute of Medicinal Biotechnology, Chinese Academy of Medical Sciences & Peking Union Medical College, Tiantan xili No.1, Beijing 100050, China.*

*To whom correspondence should be addressed.

Email: binhong69@hotmail.com; hongbin@imb.pumc.edu.cn (Bin Hong); xieyy@imb.pumc.edu.cn (Yunying Xie).

**Table of Contents**

[Table S1 Biosynthetic gene clusters identified by antiSMASH 5.0.0 in the *S. mobaraensis* US-43 draft genome 3](#_Toc17274940)

[Table S2 NMR data compound **1** (600 (^1^H) and 150 (^13^C) MHz, in CD_3_OD) compared with those of herbicidin F 4](#_Toc17274941)

[Table S3 Primers used in this study 5](#_Toc17274942)

[Figure S1 Circular genome map of *S. mobaraensis* US-43 7](#_Toc17274943)

[Figure S2 MS/MS fragments for herbicidin F 8](#_Toc17274944)

[Figure S3 EMSA analysis of HcdR2 with the postulated promoter regions of the *hcd* cluster 9](#_Toc17274945)

[Figure S4 HPLC, UV and HRMS spectra of compound **1**, **2** and **3** 10](#_Toc17274946)

[Figure S5 ^1^H NMR for **2** (herbicidin K) in DMSO-*d_6_* (600 MHz) 11](#_Toc17274947)

[Figure S6 ^1^H NMR for **3** (herbicidin O) in DMSO-*d_6_* (600 MHz) 12](#_Toc17274948)

[Figure S7 ^1^H NMR for **1** (herbicidin F) in CD_3_OD (600 MHz) 13](#_Toc17274949)

[Figure S8 ^13^C NMR for **1** (herbicidin F) in CD_3_OD (150 MHz) 14](#_Toc17274950)

[Figure S9 ^1^H NMR for **1** (herbicidin F) in DMSO-*d_6_* (600 MHz) 15](#_Toc17274951)

# Table S1 Biosynthetic gene clusters identified by antiSMASH 5.0.0 in the *S. mobaraensis* US-43 draft genome

| **Cluster** | **Type** | **Scaffold** | **From** | **To** | **Most similar known cluster** | **Similarity** |
| --- | --- | --- | --- | --- | --- | --- |
| Cluster 1 | lanthipeptide | 1 | 462,623 | 486,997 | Chejuenolide A | 7% |
| Cluster 2 | T3PKS | 1 | 802,918 | 843,979 | Siomycin | 7% |
| Cluster 3 | lassopeptide | 2 | 437,036 | 459,576 | ND |  |
| Cluster 4 | terpene | 2 | 668,364 | 689,662 | ND |  |
| Cluster 5 | saccharide | 3 | 522,945 | 606,386 | Avilamycin A | 77% |
| Cluster 6 | lanthipeptide | 4 | 34,308 | 56,947 | Informatipeptin | 42% |
| Cluster 7 | terpene | 4 | 153,541 | 174,489 | ND |  |
| Cluster 8 | PKS-terpene | 4 | 223,666 | 341,822 | Napyradiomycin | 19% |
| Cluster 9 | terpene | 4 | 348,736 | 405,977 | Hopene | 76% |
| Cluster 10 | indole | 4 | 420,217 | 443,792 | AT2433 | 14% |
| Cluster 11 | T3PKS-terpene-NRPS | 4 | 461,405 | 530,023 | Griseobactin | 23% |
| Cluster 12 | terpene | 5 | 1 | 20821 | Xiamycin | 13% |
| Cluster 13 | NRPS-T1PKS | 5 | 260,198 | 309,239 | Malleilactone | 38% |
| Cluster 14 | T1PKS | 5 | 323,034 | 370,572 | Filipin | 15% |
| Cluster 15 | T2PKS | 5 | 370,695 | 443,940 | Simocyclinone D9 | 33% |
| Cluster 16 | terpene | 5 | 477,541 | 498,467 | ND |  |
| Cluster 17 | siderophore | 6 | 49,198 | 61,150 | ND |  |
| Cluster 18 | lassopeptide | 6 | 276,280 | 298,825 | Citrulassin D | 40% |
| Cluster 19 | T1PKS-NPRS | 6 | 361,485 | 488,344 | Paulomycin | 9% |
| Cluster 20 | lassopeptide | 7 | 303,727 | 326,353 | Citrulassin B | 100% |
| Cluster 21 | T3PKS | 7 | 331,307 | 372,413 | ND |  |
| Cluster 22 | fused | 8 | 1 | 20,126 | Ketomemicin B3 | 100% |
| Cluster 23 | T1PKS | 8 | 71,197 | 214,197 | Laidlomycin | 50% |
| Cluster 24 | T1PKS | 8 | 316,310 | 354,140 | Piericidin A1 | 91% |
|  |  | 11 | 150,759 | 260,466 | Piericidin A1 | 50% |
| Cluster 25 | NPRS | 9 | 94,401 | 146,514 | Deimino-antipain | 66% |
| Cluster 26 | thiopeptide | 9 | 151,512 | 180,639 | Cyclothiazomycin B | 100% |
| Cluster 27 | T1PKS | 9 | 187,522 | 306,141 | Sceliphrolactam | 72% |
| Cluster 28 | terpene | 11 | 34,650 | 55,885 | ND |  |
| Cluster 29 | lassopeptide | 12 | 141,181 | 163,770 | Keywimysin | 80% |
| Cluster 30 | NPRS | 13 | 142,656 | 185,573 | Skyllamycin | 4% |
| Cluster 31 | T1PKS | 14 | 127,267 | 171,280 | Terfestatin | 14% |
| Cluster 32 | other | 15 | 66,924 | 108,297 | A-503083 | 9% |
| Cluster 33 | lanthipeptide | 15 | 127,835 | 151,185 | Steffimycin | 8% |
| Cluster 34 | T1PKS | 16 | 3,211 | 52,272 | Ansamitocin | 9% |
| Cluster 35 | Terpene-lanthipeptide | 17 | 2,186 | 44,461 | Kanamycin | 53% |
| Cluster 36 | thiopeptide | 17 | 65,682 | 97,094 | Sch 18640 | 9% |
| Cluster 37 | NPRS-T1PKS | 19 | 4,373 | 120,841 | Bleomycin | 96% |
| Cluster 38 | NRPS | 20 | 1 | 37,861 | Rimosamide | 35% |
| Cluster 39 | lassopeptide | 21 | 60,403 | 92,377 | ND |  |
| Cluster 40 | terpene | 22 | 2 | 16,037 | Streptomycin | 2% |
| Cluster 41 | terpene | 22 | 36,087 | 57,106 | ND |  |
| Cluster 42 | NRPS | 24 | 9,940 | 56,704 | Qinichelins | 66% |
| Cluster 43 | other | 25 | 1 | 50,707 | Pseudouridimycin | 68% |
| Cluster 44 | terpene | 28 | 1 | 13,568 | Lactonamycin | 3% |

ND: Not Detected

# Table S2 NMR data compound 1 (600 (^1^H) and 150 (^13^C) MHz, in CD_3_OD) compared with those of herbicidin F

|  | **1** | | Herbicidin F | |
| --- | --- | --- | --- | --- |
|  |  |  |  |  |
| 2 | 8.36, s | 150.3 | 8.23, s | 152.7 |
| 4 |  | 149.5 |  | 149.1 |
| 5 |  | 119.9 |  | 118.0 |
| 6 |  | 154.4 |  | 156.0 |
| 8 | 8.09, s | 141.8 | 7.97, s | 139.5 |
| 1’ | 6.09, d (1.6) | 89.1 | 6.07, d (2.0) | 87.3 |
| 2’ | 4.08, d (1.1) | 91.8 | 4.08, d (2.0) | 90.3 |
| 3’ | 4.50, d (1.5) | 74.7 | 4.51, br d (2.0) | 73.2 |
| 4’ | 4.41, q (2.4) | 79.4 | 4.41, q (2.4) | 77.5 |
| 5’ | 2.28, m | 26.7 | 2.28, m | 25.2 |
| 6’ | 4.52, dd (10.3, 5.7) | 66.7 | 4.55, dd (6.4, 14.4) | 65.2 |
| 7’ |  | 93.5 |  | 91.9 |
| 8’ | 5.00, d (3.2) | 72.0 | 5.02, d (3.2) | 70.5 |
| 9’ | 4.30, dd (3.2, 1.0) | 70.6 | 4.34, dd (1.2, 3.2) | 69.1 |
| 10’ | 4.45, s | 78.4 | 4.48, br s | 76.9 |
| 11’ |  | 171.4 |  | 169.8 |
| 1” |  | 167.2 |  | 165.7 |
| 2” |  | 128.6 |  | 127.0 |
| 3” | 6.71, q (7.1) | 142.1 | 6.73, q (7.2) | 140.3 |
| 4” | 1.90, d (7.0) | 15.2 | 1.91, q (7.2) | 13.7 |
| 5” | 1.85, s | 12.5 | 1.89, s | 10.9 |
| 2’-OCH_3_ | 3.41, s | 58.5 | 3.43, s | 56.9 |
| 11’-OCH_3_ | 3.61, s | 52.8 | 3.63, s | 51.3 |
|  |  |  |  |  |

# Table S3 Primers used in this study

| **Name** | **Sequence（5’-3’）** | **Purpose** |
| --- | --- | --- |
| pL-hcdR1-F  pL-hcdR1-R | ATCATATGGCCGCCGAACTGTCCGAC  ATAGGATCCTCAGTGCGTCGGCCTCGGTA | Used to amplify *hcdR1* |
| pL-hcdR2-F  pL-hcdR2-R | TACATATGTATTACGCAGTTCTCGGACC  GATCTAGATCAACGGACTAGTCAACGGA | Used to amplify *hcdR2* |
| pL-hcdR3-F  pL-hcdR3-R | TACATATGCTGGTCGGACGGGAGTGT  TAGGATCCCATCACCGACATCTCCCACC | Used to amplify *hcdR3* |
| pSET152  attB_streptomyces | TTCGGCGGCTTCAAGTTCGG  CGGTGGGGGTGCCAGGG | Used to verify the φC31 integration site |
| US43-hrdB-RT-F  US43-hrdB-RT-R | GACCAGATTCCGCCAACCC  CCTCTGCCGCACTGACCAT | Used to detect the *hrdB* transcription in *S. mobaraensis* US-43 |
| US43-hcdR1-RT-F  US43-hcdR1-RT-R | CACGATCTCGTGCGGCTCT  CCCGTGGCGAGGTAGTGGT | Used to detect the *hcdR1* transcription |
| US43-hcdR2-RT-F  US43-hcdR2-RT-R | GCTGGTCGGACGGGAGTG  GTACGAGGGCGTGGCTGC | Used to detect the *hcdR2* transcription |
| US43-hcdR3-RT-F  US43-hcdR3-RT-R | GAGTTTCGACTCGTGGCTG  GAAACTGATCGCCGAGTGAC | Used to detect the *hcdR3* transcription |
| US43-hcdT-RT-F  US43-hcdT-RT-R | GCGGACTGGCGATGAGCA  CGGGCAAGGCGAGAAGGTA | Used to detect the *hcdT* transcription |
| US43-hcdG-RT-F  US43-hcdG-RT-R | ACGGTGGAGACATTGAGGC  CCGGCTGCTGATCTACGAG | Used to detect the *hcdG* transcription |
| US43-hcdH-RT-F  US43-hcdH-RT-R | CGCAGGGTGCCAGGAT  CGTGGTGGGCGTGGTT | Used to detect the *hcdH* transcription |
| US43-hcdF-RT-F  US43-hcdF-RT-R | ATGGCCTCGTGCATCCT  CAGTGGTGGCAGTGGGTG | Used to detect the *hcdF* transcription |
| US43-hcdD-RT-F  US43-hcdD-RT-R | CGAACTGGCCTATTTCCACG  GAAGTCGGCGAGCTGGTC | Used to detect the *hcdD* transcription |
| US43-hcdE-RT-F  US43-hcdE-RT-R | GCCTGGACTTCGTGTTCTTC  GGTGACGTCGTTCTTGAGGA | Used to detect the *hcdE* transcription |
| US43-hcdC-RT-F  US43-hcdC-RT-R | GGGCAGTTCGGTGAGGGTG  GCATGGGGCTGTCCGTGTC | Used to detect the *hcdC* transcription |
| US43-hcdB-RT-F  US43-hcdB-RT-R | ACGTCCGCAGCAGTCCCCTGTC  CGAGGCGGTGAGCACGAT | Used to detect the *hcdB* transcription |
| US43-hcd3-RT-F  US43-hcd3-RT-R | GCGTGGGCAAGAGCCGTAC  CCGTGACGCCTCCTTGGG | Used to detect the *hcd3* transcription |
| US43-hcd2-RT-F  US43-hcd2-RT-R | GGACCTGCCGCATTACCG  CTCCACCGCCTCCCGTAA | Used to detect the *hcd2* transcription |
| US43-hcd1-RT-F  US43-hcd1-RT-R | ACCGAACCACTGCCCTACC  CTATCCCAGGTCTCGTTGTCG | Used to detect the *hcd1* transcription |
| HcdR2-16b-F2  HcdR2-16b-R2 | ATACATATGCTGGTCGGACGGGAGTGT  TATAGGATCCTCAGCCGGAGGAGCGTCC | Used for *hcdR2* expression in *E. coli* |
| R2-Bp-F  R2-Bp-R | CGATGCTCCTTTCCCTGGGC  TCCCTCGCCGGCGGACACGC | Used to amplify 303 bp of the promoter region *hcdR2-B* |
| Tp-1-F  Tp-1-R | CCGGCGGGCCCGCCCTGCGC  GATTCAGCCAAGTGACCCCG | Used to amplify 300 bp subfragment 1 of upstream region of *hcdT* |
| Tp-2-F  Tp-2-R | GCGCCGTCGCGCATCGTCAA  CACTGTGGATGCCATGGAAG | Used to amplify 476 bp subfragment 2 of upstream region of *hcdT* |
| R1p-F  R1p-R | GCTGCGCGACGGCTGGCC  CGCGCCCAGCGGCCAGGT | Used to amplify 101 bp upstream region of *hcdR1* |
| her3p-F  her3p-R | CATGAAGGCCGGCACTTTG  ATCCAACCCCTAAGACACCG | Used to amplify 326 bp upstream region of *her3* from *Streptomyces* sp. L-9-10 |
| V2-Bp-F  V2-Bp-R | CTCTTCGCGGGGAGAGAG  AACCTCTTTCTCGTCGTGGA | Used to amplify 284 bp upstream region of *hcdB* homologous gene from *Streptomyces* sp. V2 |

# Figure S1 Circular genome map of *S. mobaraensis* US-43

Circos plot showing the draft genome of *S. mobaraensis* US-43, BGCs predicted by antiSMASH, GC content and skew, as well as the BGCs of herbicidin F, piericidin A and isocoumarins which have been isolated from the wild strain of *S. mobaraensis* US-43

**
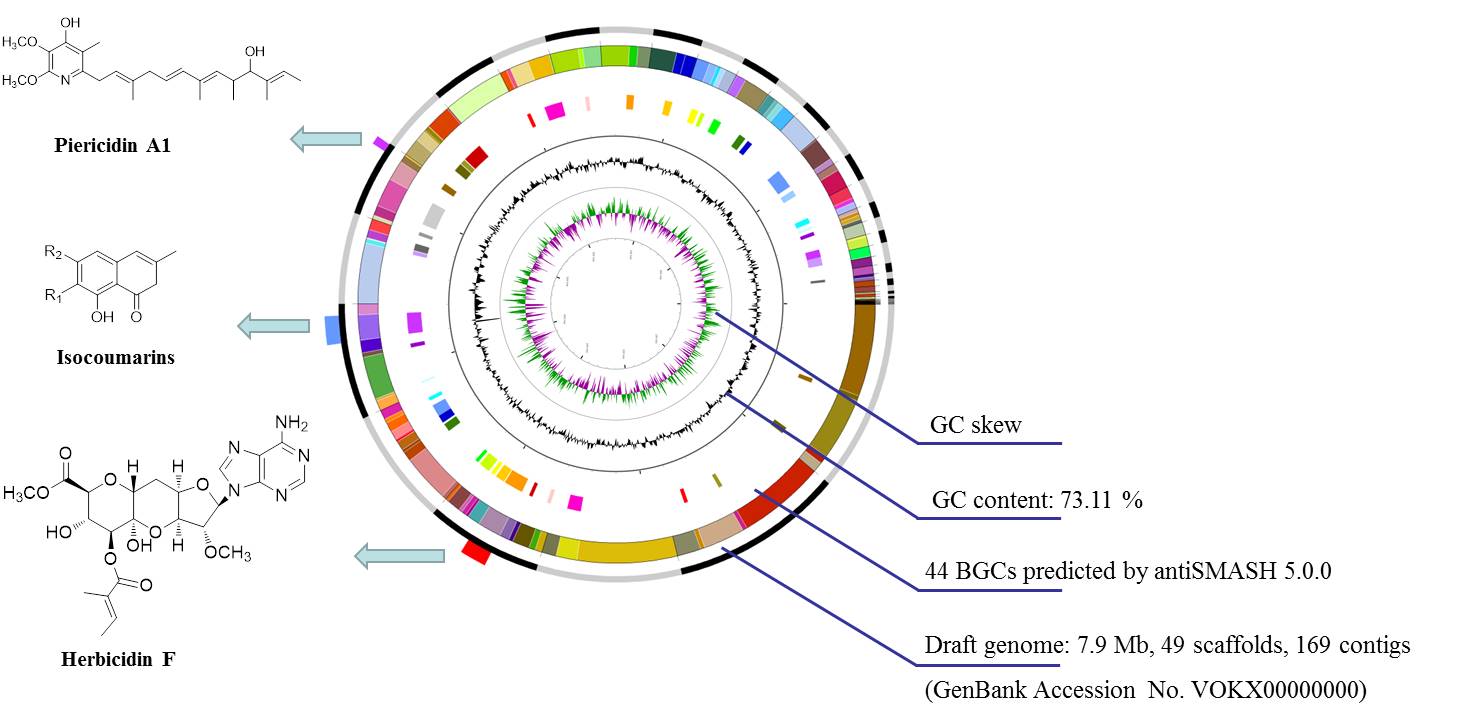
**

# Figure S2 MS/MS fragments for herbicidin F

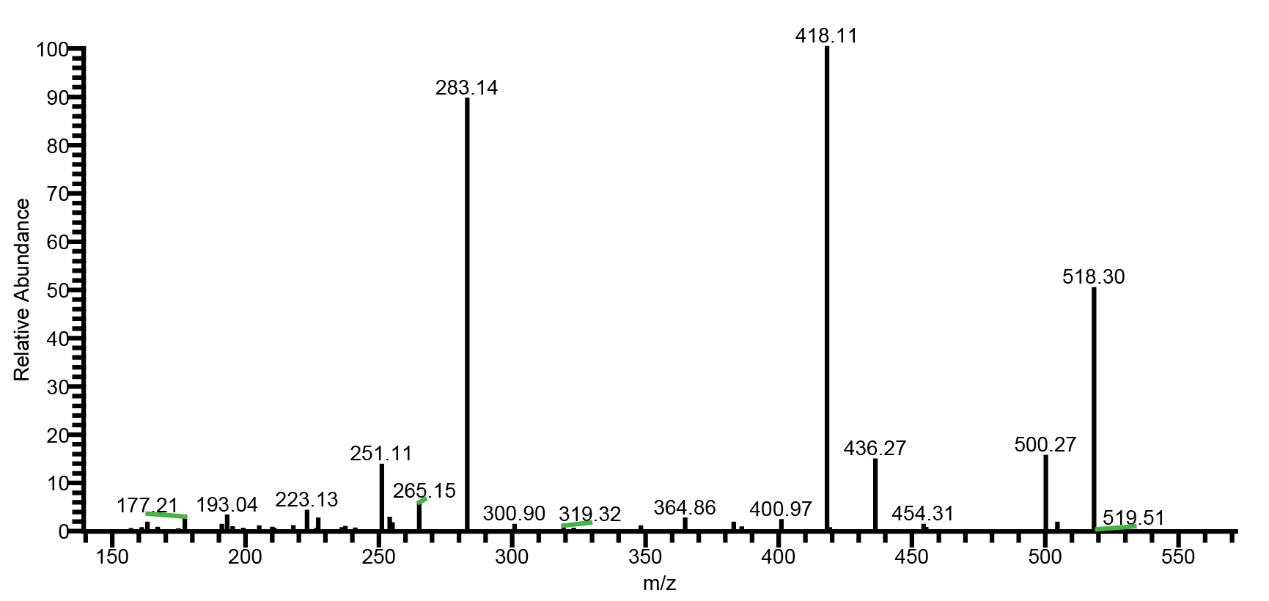


# Figure S3 EMSA analysis of HcdR2 with the postulated promoter regions of the *hcd* cluster

(A) Purification of the His_10_-HcdR2 from *E. coli* BL21(DE3). Lane 1-3, purified His_10_-HcdR2. (B) EMSA analysis of 5’ biotin labeled *hcdT-1*p with HcdR2.


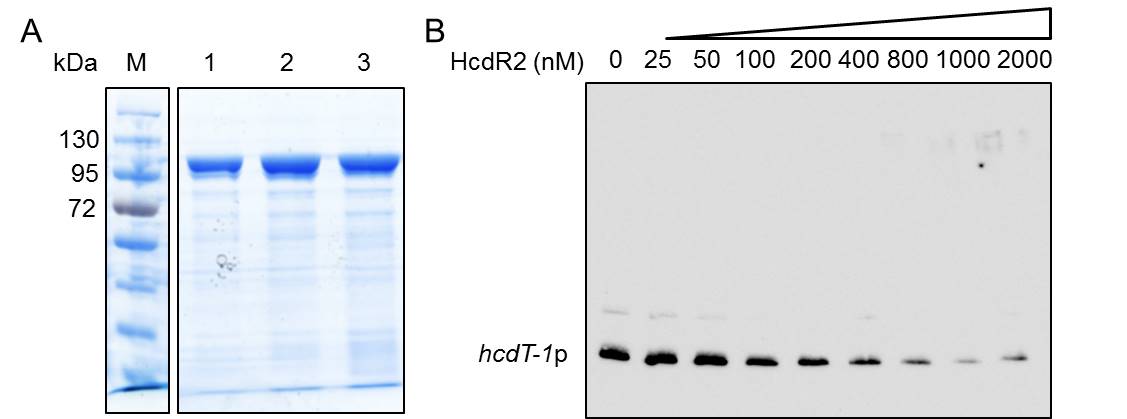


# Figure S4 HPLC, UV and HRMS spectra of compound 1, 2 and 3


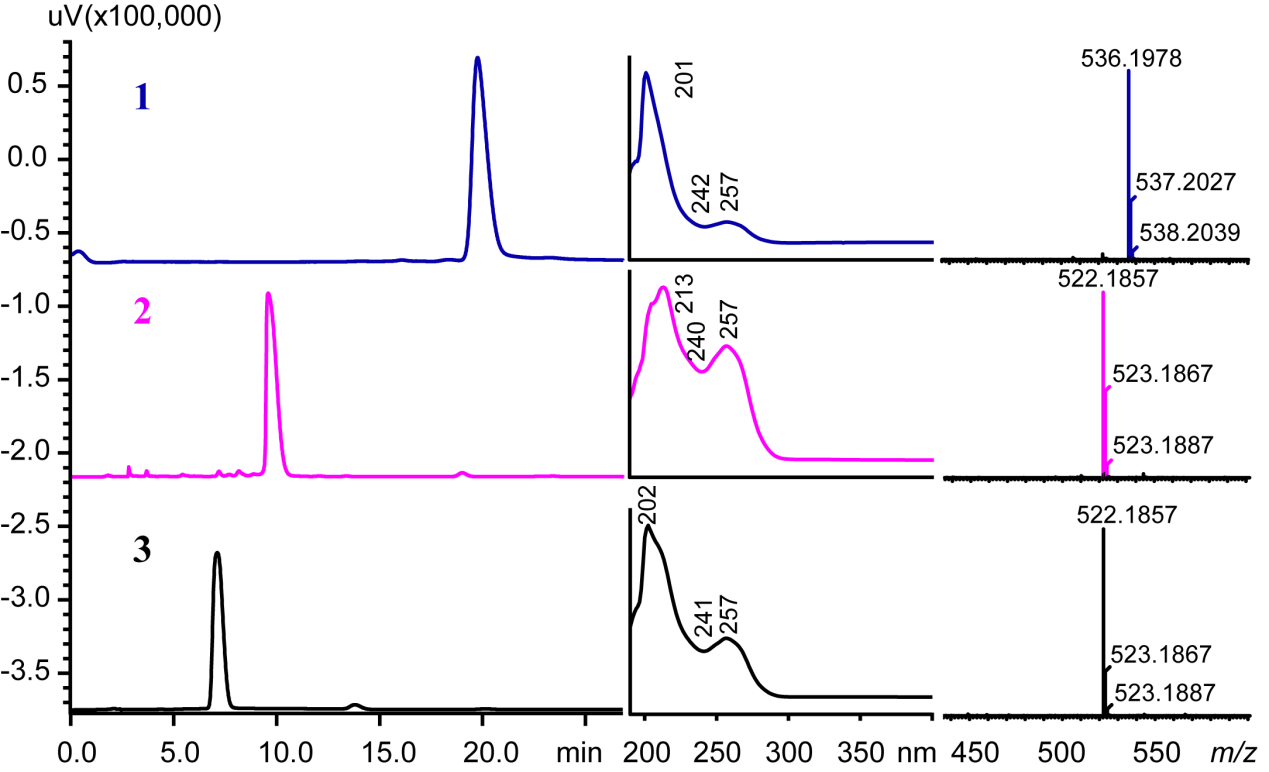


# Figure S5 ^1^H NMR for 2 (herbicidin K) in DMSO-*d_6_* (600 MHz)





# Figure S6 ^1^H NMR for 3 (herbicidin O) in DMSO-*d_6_* (600 MHz)





# Figure S7 ^1^H NMR for 1 (herbicidin F) in CD_3_OD (600 MHz)

# Figure S8 ^13^C NMR for 1 (herbicidin F) in CD_3_OD (150 MHz)

# Figure S9 ^1^H NMR for 1 (herbicidin F) in DMSO-*d_6_* (600 MHz)
